# Preparing Future Physicians to Address the Social Needs of Patients in Their Daily Clinical Practice: An Interactive Workshop

**DOI:** 10.15766/mep_2374-8265.11595

**Published:** 2026-04-21

**Authors:** Hejin Jeong, Patricia Kachmyers, Ifeolorunbode A. Adebambo

**Affiliations:** 1 Medical Student, Case Western Reserve University School of Medicine; 2 Social Work Supervisor, Department of Social Work, MetroHealth Medical Center; 3 Family Medicine Specialist, Department of Family Medicine, MetroHealth Medical Center; Clinical Associate Professor, Case Western Reserve University School of Medicine

**Keywords:** Health Disparities, Patient-Centered Care, Communication Skills, Diversity, Equity, Inclusion, Social Determinants of Health, Case-Based Learning

## Abstract

**Introduction:**

Despite the growing emphasis on social determinants of health (SDH) education in medical schools, clinicians still infrequently screen for and address SDH. We developed an interactive and skills-focused workshop to enhance medical students’ confidence and ability to address SDH.

**Methods:**

Preclerkship medical students engaged in a 90-minute workshop. The workshop offered a case-based didactic and a small-group patient interview simulation experience to foster students’ ability to employ validated SDH screening tools, devise management plans, connect patients to social workers and resources, and document SDH using diagnostic codes. Pre-, post-, and 1-year follow-up surveys were administered to assess the efficacy of this intervention.

**Results:**

Twenty-five students (58.3% first-year students; 70.8% female) completed pre- and postsurveys; 24 completed the follow-up survey. Students found the workshop engaging and particularly valued the small-group simulation and education on social workers and SDH documentation (>85% positive ratings). Postworkshop, students showed improved knowledge, confidence, and attitudes toward addressing SDH (*P* < .0001, effect size > 0.85), with a sustained increase in confidence and frequency of SDH screening after 1 year (*P* < .05). Qualitative feedback confirmed greater attention to SDH and biases in patient care and increased appreciation for social workers, but also highlighted a need for increased opportunity for continued reinforcement and applied learning.

**Discussion:**

A simulation-based workshop can provide an easily scalable method to equip medical students with the practical skills needed to address SDH in clinical practice. Its low logistical requirements make it well suited for institution-wide adoption across large academic institutions.

## Educational Objectives

By the end of this activity, learners will be able to:

1. Describe the main domains of social determinants of health.
2. Identify the common practical challenges that physicians currently face in addressing patients’ socioeconomic needs in their daily practice.
3. Use a validated social needs screening tool to efficiently identify subtle socioeconomic factors contributing to medical issues within the time constraints of a typical patient encounter.
4. Navigate patient referral resources available within their institution and the local community.
5. Devise an actionable management plan to address a patient's socioeconomic needs.
6. Outline the roles of social workers in addressing patients’ socioeconomic needs.
7. Explain the importance of documenting patients’ socioeconomic needs in the electronic medical record system using appropriate diagnostic codes.

## Introduction

Despite advances in medical science, certain US populations experience worse health outcomes than others, which are significantly driven by social determinants of health (SDH).^1–3^ Although many medical education curricula include SDH education,^4,5^ physicians still infrequently screen for or address patients’ social needs.^6,7^ Time constraints, physicians’ perceived lack of knowledge and confidence, and limited reimbursement are major barriers to addressing SDH,^6–10^ pointing to a gap between classroom instruction and the realities of clinical practice. For instance, we identified several limitations of the core SDH education curriculum at our institution, Case Western Reserve University School of Medicine (CWRUSOM),^11^ that, according to our literature review, are also prevalent across other medical schools.^12^

Currently, how medical schools approach SDH training is suboptimal. At CWRUSOM, education specifically focused on SDH is oftentimes front-loaded in the first few weeks of medical school curricula,^12,13^ when students are unfamiliar with clinical workflows, diagnostic reasoning, and care planning. Consequently, instruction is primarily limited to the theoretical concept of SDH and their influence on health outcomes, with weaker emphasis on developing skills to comprehensively screen for SDH beyond modifiable risk factors and occupational hazards within patient encounters, or on tailoring actionable plans to address them.^5,12,13^ Additionally, because SDH is largely taught in lecture- or discussion-based formats, students lack structured opportunities to practice identifying and managing SDH within a typical patient encounter that simulates real clinical practice settings. More experiential learning is largely confined to elective courses or tracks, which can accommodate only a limited number of students and tend to attract those already interested in SDH.^5,13^ Moreover, while an interprofessional preclerkship course was recently piloted, the content centered on improving transitions of care in biomedical contexts, with minimal education on collaborating with social workers (SWs) to address SDH that often complicates care; this gap extends into the clerkship years, during which students rarely interact with SWs.^14,15^ Lastly, current curricula offer little to no training on the value of systematically documenting SDH, such as through the International Statistical Classification of Diseases and Related Health Problems (ICD) Z-codes.^12^ The resulting lack of physician trainees’ awareness may perpetuate the current underdocumentation of SDH,^16^ which undermines clinicians’ ability to tailor patient care, opportunities for quality improvement initiatives by obscuring the population-level burden of SDH, and reimbursement rates for addressing SDH.^16,17^

Studies have shown promising educational outcomes following various interventions such as service-learning or home-visit programs.^18–20^ However, consistent with the general trend of current UME curricula, these interventions were largely conducted as elective courses, likely due to the logistical difficulty of securing the curricular space, patient and community partnerships, faculty commitment,^20^ and administrative support,^5,19^ which hinder wider implementation of these types of intervention, especially at large institutions.^12^ Furthermore, although a few interventions specifically targeting SW education in UME have been developed,^14,21^ the SW component is often overlooked in intervention efforts broadly aimed at enhancing SDH education.^18–20^ Likewise, systemic SDH documentation is rarely included. To fill these gaps, we designed a pilot educational workshop that can be easily delivered in the classroom and scaled to fit larger institutional curricula. Widespread adoption of this curriculum across multiple institutions could serve as a catalyst for a broader shift in medical education, ultimately empowering the next generation of physicians to navigate the social complexities in modern medicine that compromise patient health outcomes.

## Methods

We utilized Kern's 6-step approach^22^ to design this workshop, as summarized in Table 1. Problem identification began with 1 author's (Hejin Jeong) personal experience as a CWRUSOM student, which prompted a review of the current SDH curriculum and the literature. We then surveyed current CWRUSOM students to conduct a needs assessment and confirm Hejin Jeong's perception of potential opportunities to improve CWRUSOM's SDH curriculum. With guidance from experts in medical education curriculum design, education evaluation and research, and preventive medicine and social work, we defined our educational objectives (EOs), implementation plan, and evaluation methodsn.

**Table 1.**
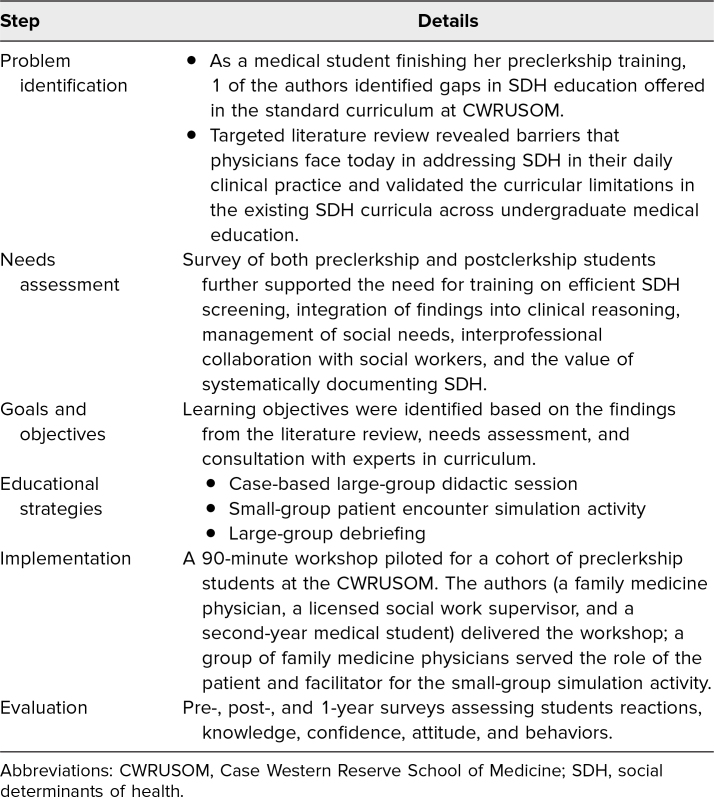
Summary of the Education Intervention Design Process Based on the Six-Step Model of Curriculum Development

### Participants and Setting

We invited first- (M1) and second-year (M2) CWRUSOM students to participate in an optional 90-minute workshop in January 2024. Students’ participation in the workshop did not affect their grades. Students received a $20 gift card after completing the presurvey, attending the workshop, and completing the postsurvey. Those who completed the 1-year follow-up survey were additionally compensated with a $10 gift card, for a total of $30. We did not provide students with any preparation materials besides the workshop EOs and agenda. This project received approval from the Case Western Reserve University Institutional Review Board (approval number: STUDY20230907).

We focused on preclerkship students for several key reasons. First, because most medical schools cover SDH education in the preclerkship years,^4^ and since these years tend to offer more flexibility compared to clerkship years, an intervention targeting preclerkship students may be more practical for other institutions to adopt. Second, because preclerkship students are still in the foundational stages of their training, early exposure to SDH could present a unique opportunity to shape their attitudes and behaviors toward integrating SDH into their learning and clinical practice as they advance through their education. Finally, the preclinical setting may be more conducive to learning foundational principles and skills in SDH, as students often have more time to engage with the material and address skill gaps before entering the clerkship years, when they face higher academic demands and fast-paced clinical environments.

### Workshop Design

The workshop consisted of an initial case-based didactic, a small-group simulation experience with a standardized patient, and a final large-group reflective discussion. Considering that current research on the best pedagogical methods to teach SDH is limited due to the heterogeneity among SDH curricula,^5,12,13^ we selected methods based on Bloom's Taxonomy^23^ and their suitability for large-scale classroom delivery. Reflective discussion was included because critical reflection has been recognized as an essential element of experiential learning for shifting students’ attitudes toward vulnerable individuals.

#### Case-based didactic (Remember and Understand levels of Bloom's Taxonomy)

The authors delivered the didactic session to orient students to the challenges of addressing social needs in clinical care, introduce validated SDH screening tools—such as the Health Leads Social Determinants of Health Screening Questions^24^ (Appendix A)—and outline pathways to connect patients with appropriate social support resources. As interdisciplinary care with SWs is a significant component of addressing SDH, a licensed SW (Patricia Kachmyers) explained the role of SWs in patient care and how students can facilitate patient referrals to SWs. Finally, we highlighted the utility of diagnostic Z-codes for documenting social needs in the electronic medical record. Appendix A includes student handouts summarizing common social support resources, and Appendix B contains presentation slides.

#### Small-group simulation Apply, Analyze, Evaluate, *and* Create *levels*

Following the didactic session, we divided students into groups of 6 or 7 for the patient interaction simulation. Students in each group collectively acted the role of a physician to interview a facilitator, who served the role of a patient. While we provided the students with basic biomedical information about the patient and the reason for the visit (Appendix C), students were responsible for identifying the patients’ additional biopsychosocial needs contributing to their medical condition by applying the strategies taught in the didactic session and the screening tool^23^ *(Apply, Analyze,* and *Evaluate)* (Appendix A). The facilitators received a 30-minute in-person training in advance, during which we oriented them to the goals and structures of the workshop and the small-group simulation, their roles, the duration of the activity, and the full details of the patient, and answered their questions. Further details of the training and the full patient vignette are described in Appendix D. After the interview, students in each small group devised a biopsychosocial problem list *(Create)* and the top 3 management steps to address the patient's needs *(Evaluate)*.

#### Reflective discussion

Students then reconvened for a brief large-group discussion, during which each small group shared their biopsychosocial problem lists and management plans. The workshop concluded with a discussion of examples of the recommended management plans for the patient case and a reflection on the small-group activity.

### Evaluation

To assess the educational efficacy of the workshop, we invited students to complete surveys that the authors developed based on the Kirkpatrick Evaluation Model^25^ before (Appendix E), immediately after (Appendix F), and 1 year after (Appendix G) the workshop.

We assessed students’ reactions (Kirkpatrick level 1) to the workshop via both Likert-style and free-response questions on the survey. We assessed students’ learning (Kirkpatrick level 2) by comparing post- and follow-up survey responses with presurvey responses. More specifically, we measured the change in knowledge by comparing students’ scores across surveys based on the answer key (Appendix H), and changes in students’ confidence in and attitude toward addressing SDH by comparing students’ responses to relevant Likert-style questions. We assessed changes in students’ behaviors (Kirkpatrick level 3) via both Likert-style and free-response questions on the follow-up survey that asked students to reflect on how the workshop influenced their clinical approach and patient interactions over the year following the workshop.

We utilized REDCap (Research Electronic Data Capture)^26^ to collect survey responses.

### Statistical Analysis

We conducted all statistical analyses using R version 4.3.3. We assigned ordinal scales to compute Likert-style responses and paired the pre-, post-, and 1-year follow-up surveys using unique student identification numbers. To evaluate the statistical significance of changes in knowledge scores, confidence, and attitude, we conducted a paired Wilcoxon signed-rank test with Pratt^27^ correction, a nonparametric test well suited for small, paired ordinal data that increases sensitivity by accounting for zero-difference ties without assuming normality. A 2-tailed *P* value < .05 was considered statistically significant. To support interpretation beyond statistical significance, we quantified the effect size by calculating the Rosenthal correlation coefficient and its 95% CI via bootstrap resampling.

## Results

Twenty-five preclerkship students (58% M1; 71% female) completed the presurvey and the postsurvey. Twenty-four students (54% M1; 75% female) completed the 1-year survey.

### Students’ Reactions

The postsurvey revealed that students had an overall positive reaction to the workshop experience, with nearly all students (92%) expressing interest in attending a follow-up session (Table 2). Students indicated that the workshop achieved its 8 EOs with 72%–96% of students rating each objective as offering moderate or significant added educational value beyond those offered by the existing preclerkship curriculum (see Table 2). Both Likert survey data and free-response feedback highlighted that presentations on documentation and SWs were uniquely valuable, with students advocating for further education in these areas. Students found the small-group component significantly more engaging than the large-group sessions, as reflected in nearly unanimous positive Likert ratings. Free-response feedback reinforced this preference, with students repeatedly highlighting the value of this active-learning simulation format and recommending even smaller group sizes. Furthermore, students appreciated the focus on actionable steps and desired additional stepwise guidance on accessing specific social resources and contacting SWs.

**Table 2.**
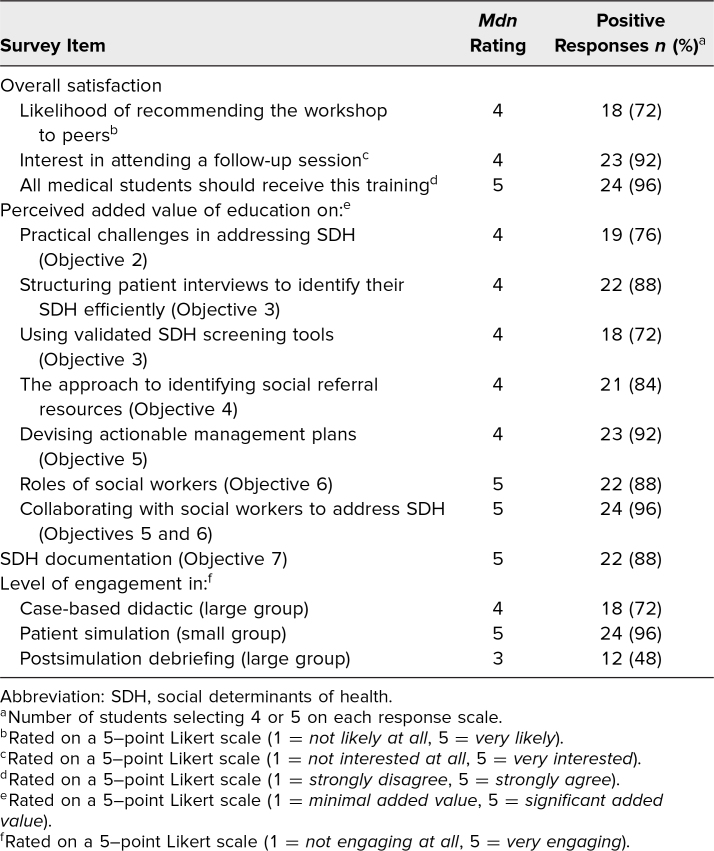
Students’ Postsurvey (*N* = 25) Responses

### Students’ Learning

Compared to baseline, students reported significantly higher confidence in the targeted workshop skills (EO#3-6) in both the post- and 1-year surveys (Table 3). In all 3 surveys, students performed well on the knowledge test (EO 1), with average scores of 83% (*n* = 25, *SD* = 8%, *P* < .01) at the immediate postsurvey and 80.3% (*n* = 24, *SD* = 7%, *P* = .31).

**Table 3.**
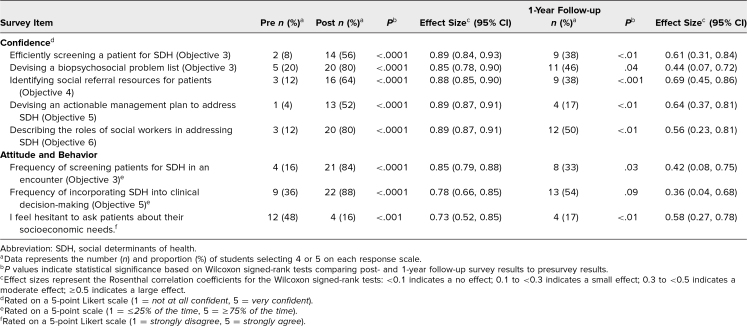
Proportions of Positive Likert Responses to the Confidence-, Attitude-, and Behaviors-Related Questions on the Pre-/Post- (*N* = 25) and 1-Year Follow-up (*N* = 24) Surveys

### Students’ Behaviors

In both the post- and 1-year surveys, students reported significantly reduced hesitancy to screen for SDH and increased screening frequency for SDH (EO 3) (see Table 3). While students had an increase in the perceived frequency of incorporating SDH into clinical decision‑making (EO 5) on the postsurvey, the increase in reported behavior at 1 year did not reach statistical significance (*P* = .09; see Table 3). The effect sizes of the changes observed in the 1-year survey were smaller than those in the postsurvey (see Table 3).

At 1 year, the majority agreed or strongly agreed that the workshop had a positive impact on their patient interactions (67%), helped them pay closer attention to SDH (54%), and that they applied a skill or concept learned in the workshop (50%; Table 4). In their qualitative feedback, students highlighted that they gained deeper awareness of structural barriers, which kept their “biases in check when interacting with patients,” and felt comfortable engaging with SWs and available social resources, making them “more likely to bring [them] up [to] the team” during rounds. Conversely, some noted that limited interactions with SWs and reinforcement constrained long-term impact.

**Table 4.**
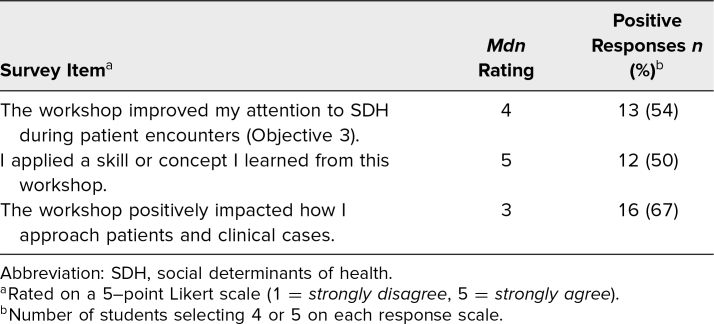
Students’ 1-Year Follow-up (*N* = 24) Responses

## Discussion

Despite growing recognition of SDH in medical education, current curricula often struggle to translate SDH knowledge into clinical practice.^5,28^ This often leaves trainees unprepared to integrate SDH screening into their clinical conceptualization and management plans, as evidenced by the discrepancy we observed between their strong baseline performance on the theoretical knowledge test and their low confidence in the key skills essential to addressing SDH.

Addressing SDH in real-world clinical practice is a complex challenge that requires structured training beyond conceptual education, which is logistically difficult to implement widely in UME.^12^ Nonetheless, results of our workshop implementation demonstrated that, even with minimal curricular time and administrative resources, a skills-based workshop can significantly enhance students’ confidence in comprehensively screening for SDH, integrating such information into clinical reasoning and decision making to address the biopsychosocial origins of patients’ presenting complaints, and navigating social support resources. Beyond these skills, the workshop elicited lasting shifts in students’ attitudes and behaviors toward integrating social contexts of patients in their clinical workflow, with heightened awareness of the social and structural barriers influencing patient care, as well as their own biases and assumptions during patient interactions.

Given the ease of scalability of our intervention, a multi-institutional adoption of this workshop may have the potential to eventually counter the trend of low SDH screening rates and the poor clinician attitudes seen today.^6,7^ In the following, we highlight additional insights based on our results that could guide educators seeking to strengthen their SDH curriculum.

### Curriculum Organization

As our students consistently perceived the small-group patient interview activity to be the most effective and engaging learning format, we recommend maximizing small-group simulation-based learning, ideally with fewer than 6 students per group. While some didactic components may be initially necessary to adequately equip students with the foundational knowledge needed for hands-on practice sessions, the reflection session following the patient interview activity could be achieved within small groups rather than reconvening as a group. Alternatively, the reflection component could be delivered via direct dialogue sessions with patient guest speakers to allow students to compare the perspectives they formed in their physician roles to patients’ lived experience of navigating social barriers.

Furthermore, student feedback indicated a clear desire for more granular education on social resources. While our workshop introduced these concepts and some examples, such as the 340B Program (Appendix B), the time constraints of a single-session workshop limited the depth of our instruction on a single topic. Therefore, while a single-session design can minimize the additional curricular space required, which is often constrained in already tightly packed UME curricula,^19^ given the breadth and the complexity of skills required in addressing SDH in patient care, we recommend expanding this intervention into a series of workshops that each delve deeper into specific concepts or skills introduced in this pilot workshop. This multisession design would allow for the inclusion of more diverse, concrete examples of common social resources (eg, Medicaid, welfare programs), their basic eligibility determination workflows, and common barriers that patients face when accessing these resources. As exemplified by the intervention of Burke et al.^18^ on social resource education, introducing these specific resources may be critical for helping students build a repertoire of referral options they can utilize as future physicians. In addition, this structure would help maximize active learning time per session, as a narrower focus would require less didactic instruction time to cover basic concepts before hands-on practice sessions.

Lastly, we recommend distributing the workshop sessions over multiple years to optimize the long-term educational benefit. When comparing students’ attitudes before and after the workshop, positive effects observed at the immediate postworkshop survey diminished by 1 year (Table 3), with students highlighting limited opportunities to reinforce the concepts learned in the workshop. Adopting longitudinal designs, as exemplified by previous interventional studies, along with consistent modeling by preceptors and routine incorporation of SDH screening, management, and documentation during clinical years, would support continued reinforcement needed for lasting behavioral change.^5,20,29^

### Curriculum Content

In addition to shifting the educational focus from the theoretical concept of SDH to a stepwise practical workflow of addressing SDH, we recommend incorporating interprofessional social work perspectives into the curriculum, as students identified this as a particularly valuable component of the workshop. Although the landscape of medical education has increasingly recognized the importance of interprofessional education (IPE) in recent years, SW roles are significantly underrepresented or marginalized in current UME IPE curricula compared to professionals providing medical care, such as nurses, physician assistants, and pharmacists.^15^ Increasing SW representation in medical education will not only foster the interprofessional skills necessary for effective team dynamics in collaborative practice but also provide long-term value for students in environments without dedicated social support. By internalizing social work's specialized expertise early in their medical training, students gain unique competencies in managing social risk factors that are largely absent from traditional medical training. For optimal engagement, future iterations of the workshop's SW component may be delivered as an interdisciplinary, patient-based simulation where medical and social work students jointly support patients in navigating social needs.^21^ Depending on faculty availability, personal preceptorship with SW mentors could also bolster long-term educational benefits.^12,29^

Education on SDH-related Z-codes was also well received, with many students identifying it as a novel area of training. This underscores the importance of incorporating SDH diagnostic coding into medical curricula; using electronic medical record–based simulation to teach these skills may help mitigate current underdocumentation and facilitate the data-driven interventions and care coordination necessary to advance health equity.^16,17^ However, it must be acknowledged that the extent to which students at this early stage of medical training can truly benefit from learning this topic may be debatable. Documenting SDH may also risk harming patient care by provoking negative implicit bias in other clinicians whom patients subsequently encounter.^30^ Nevertheless, given its important benefits,^16,17^ extending this training—especially for residents and fellows, for whom diagnostic coding is more immediately relevant to routine clinical work—may be a worthwhile area for continued exploration if coupled with explicit emphasis on the ethical implications of coding, patient confidentiality, transparency, and consent on documenting SDH, and sensitive coding tailored to each patient's circumstances.^30^

### Limitations

Because voluntary participation in this optional workshop could have introduced self-selection bias, confirmation of our results requires replication in larger cohorts using random sampling. Our analytical design for evaluating the workshop's efficacy in achieving its EOs largely relied on indirect measures and self-reports, which do not necessarily reflect the true frequency or improvement in the quality of students’ patient care. Therefore, institutions implementing our intervention would benefit from incorporating objective evaluation measures, such as analyses of biopsychosocial problem lists and management plans generated by student groups during patient encounter simulations. This would be particularly valuable for tracking longitudinal improvement if the intervention is delivered as a series or if students’ assessment and plan notes from subsequent clerkship years can additionally be compared. Objective behavioral assessment by qualified faculty would additionally strengthen the validity of intervention outcomes.

Similarly, although we explicitly phrased the survey questions to prompt students to reflect on the perceived impact of the workshop itself, the absence of a control group limits our ability to definitively attribute the observed improvements to the workshop rather than subsequent clinical training. This confounding is critical for the M2 cohort, whose matriculation into clinical rotations by 1 year likely offered greater real-world exposure to SDH. Because our intervention's nature as a single-session pilot workshop with a small cohort limited the statistical power required to stratify the analysis by year, multiyear replication with larger cohorts is warranted to assess this potential confounding effect and longitudinal reproducibility of our workshop.

While the shortcomings of SDH education curricula that we identified are common across US medical schools,^5,12,13,15,28^ variations among individual institutions’ curricula, students’ baseline competencies in SDH, curricular space, resources, culture, and curricular evolution may limit the interinstitutional and temporal generalizability of the efficacy of our intervention.

Because this workshop was delivered in a classroom setting, positive changes seen after this intervention may not necessarily translate to real clinical settings, where additional challenges, such as competing priorities and a demanding environment, may hinder students’ ability to routinely address SDH. Thus, simulation-based education targeting such specific barriers should be considered to better prepare future physicians to address SDH in their routine clinical practice.

## Appendices


Student Handouts.pdfIncorporating SDH Into Patient Care.pptxSmall-Group Case (Student Version).docxSmall-Group Facilitator Training and Full Vignette.docxPresurvey.docxPostsurvey.docx1-Year Follow-Up Survey.docxKnowledge Questions - Answer Key.docx
*All appendices are peer reviewed as integral parts of the Original Publication.*
