# Supplementary material for: Preparing Future Physicians to Address the Social Needs of Patients in Their Daily Clinical Practice: An Interactive Workshop
Source: MedEdPORTAL. 2026 Apr 21;22:11595. doi: 10.15766/mep_2374-8265.11595 (PMC13098288; doi:10.15766/mep_2374-8265.11595)
Supplement: Supplementary file 1 — Student Handouts.pdfIncorporating SDH Into Patient Care.pptxSmall-Group Case (Student Version).docxSmall-Group Facilitator Training and Full Vignette.docxPresurvey.docxPostsurvey.docx1-Year Follow-Up Survey.docxKnowledge Questions - Answer Key.docx [file mep_2374-8265.11595-s001.zip › A. Student Handouts.pdf]

Appendix A. Student Handouts

These printed handouts are to be distributed to the students at the beginning of the workshop.

The Health Leads Social Determinants of Health Screening Questions is provided on page 4 of this appendix for distribution along with the workshop handouts on pages 2-3.

Resources to Address Social Determinants of Health in Northeast Ohio

Social & Community

- Senior Centers
- Neighborhood or Settlement Houses
- Legal Aid

Economic Stability

- Job and Family Services benefits
- Greater Cleveland Food Bank
- Youth Opportunities Unlimited

Education Quality/Access

- Project LEARN
- College Now
- Spanish-American Committees

Healthcare Quality/Access

- Medicaid
- RTA Paratransit
- 340B Program

Neighborhood & Built Environment

- CMHA/EDEN Inc
- HEAP/PIPP
- City- and County-level senior housing improvement programs

- <u>CMHA (Cuyahoga Metropolitan Housing Authority) and EDEN Inc.</u>
  Local organizations that manage rent subsidy programs or provide housing support for individuals facing housing insecurities or homelessness
- <u>RTA (Regional Transit Authority) Paratransit</u>
  A local public transit agency's service that provides individualized rides for those who are unable to use public transportation due to disabilities
- <u>HEAP (Home Energy Assistance Program) / PIPP (Percentage of Income Payment Plan; state-level)</u>
  Federally funded (HEAP) or state-level (PIPP) utility assistance programs that help cover electricity and gas bills

Content by Patricia Kachmyers (author) and Anna Brandt, used with permission. Images by Piktochart, retrieved from: https://piktochart.com/on November 25th, 2023. Creative Commons License associated: Pexels License (similar to Creative Commons Zero (CC0): https://support.piktochart.com/article/673-citation)

Providing Support to Patients

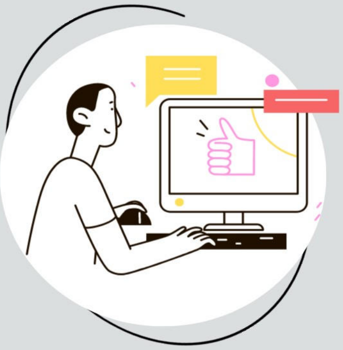

Concern comes to social worker's attention

- Provider referral
- Walk-in
- Self-referral
- Preventive screening tools
- Community events

Social worker gathers information

- Interview with patient/family
- Chart review
- Other sources as needed
  - Provider, other social workers, available documentation

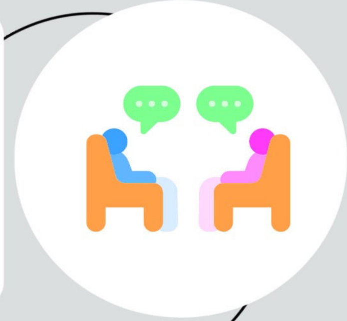

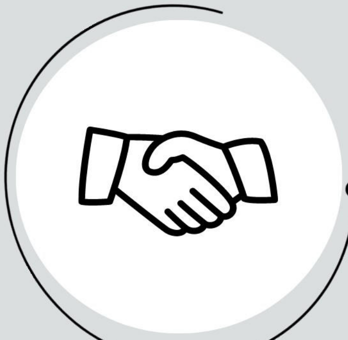

Implementation of the intervention

- Help patient understand available assistance
- Provide referral information
- Assist in calling orgs
- Assist in completing applications
- Ongoing emotional support

Reassessment and follow-up

- Did the intervention meet the need?
- Does the patient need more support in the process?

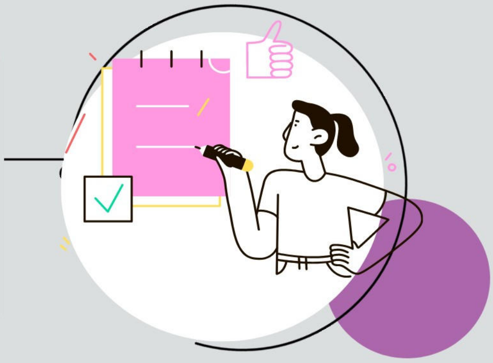

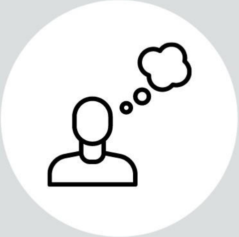

Keep in mind...

Assessment of the patient and circumstances is ongoing and may require a shift in approach or referrals

Content by Patricia Kachmyers (author) and Anna Brandt, used with permission.
Images by Piktochart, retrieved from: https://piktochart.com/on November 25th, 2023. Creative Commons License associated: Pexels License (similar to Creative Commons Zero (CC0): https://support.piktochart.com/article/673-citation)

Social Determinants of Health Screening Questions

(by Health Leads)

		Yes / No
	In the last 12 months*, did you ever **eat less than you felt you should** because there wasn't enough money for food?	Y N
	In the last 12 months, has the **electric, gas, oil, or water company threatened to shut off your services** in your home?	Y N
	Are you worried that in the next 2 months, you **may not have stable housing?**	Y N
	Do problems getting **child care make it difficult for you to work** or study? *(leave blank if you do not have children)*	Y N
$	In the last 12 months, have you needed to see a doctor, **but could not because of cost?**	Y N
	In the last 12 months, have you ever had to go without health care because you didn't have **a way to get there?**	Y N
	Do you ever need help **reading hospital materials?**	Y N
	I often feel that **I lack companionship.**	Y N
	**Are any of your needs urgent?** For example: I don't have food tonight, I don't have a place to sleep tonight	Y N
	If you checked YES to any boxes above, **would you like to receive assistance** with any of these needs?	Y N

Content by Health Leads, retrieved from: https://healthleadsusa.org/wp-content/uploads/2023/05/Screening_Toolkit_2018.pdf on November 4th, 2023. Creative Commons License associated: https://creativecommons.org/licenses/by-sa/4.0/.
