# Supplementary material for: Preparing Future Physicians to Address the Social Needs of Patients in Their Daily Clinical Practice: An Interactive Workshop
Source: MedEdPORTAL. 2026 Apr 21;22:11595. doi: 10.15766/mep_2374-8265.11595 (PMC13098288; doi:10.15766/mep_2374-8265.11595)
Supplement: Supplementary file 1 — Student Handouts.pdfIncorporating SDH Into Patient Care.pptxSmall-Group Case (Student Version).docxSmall-Group Facilitator Training and Full Vignette.docxPresurvey.docxPostsurvey.docx1-Year Follow-Up Survey.docxKnowledge Questions - Answer Key.docx [file mep_2374-8265.11595-s001.zip › B. Incorporating SDH Into Patient Care.pptx]

## Slide 1
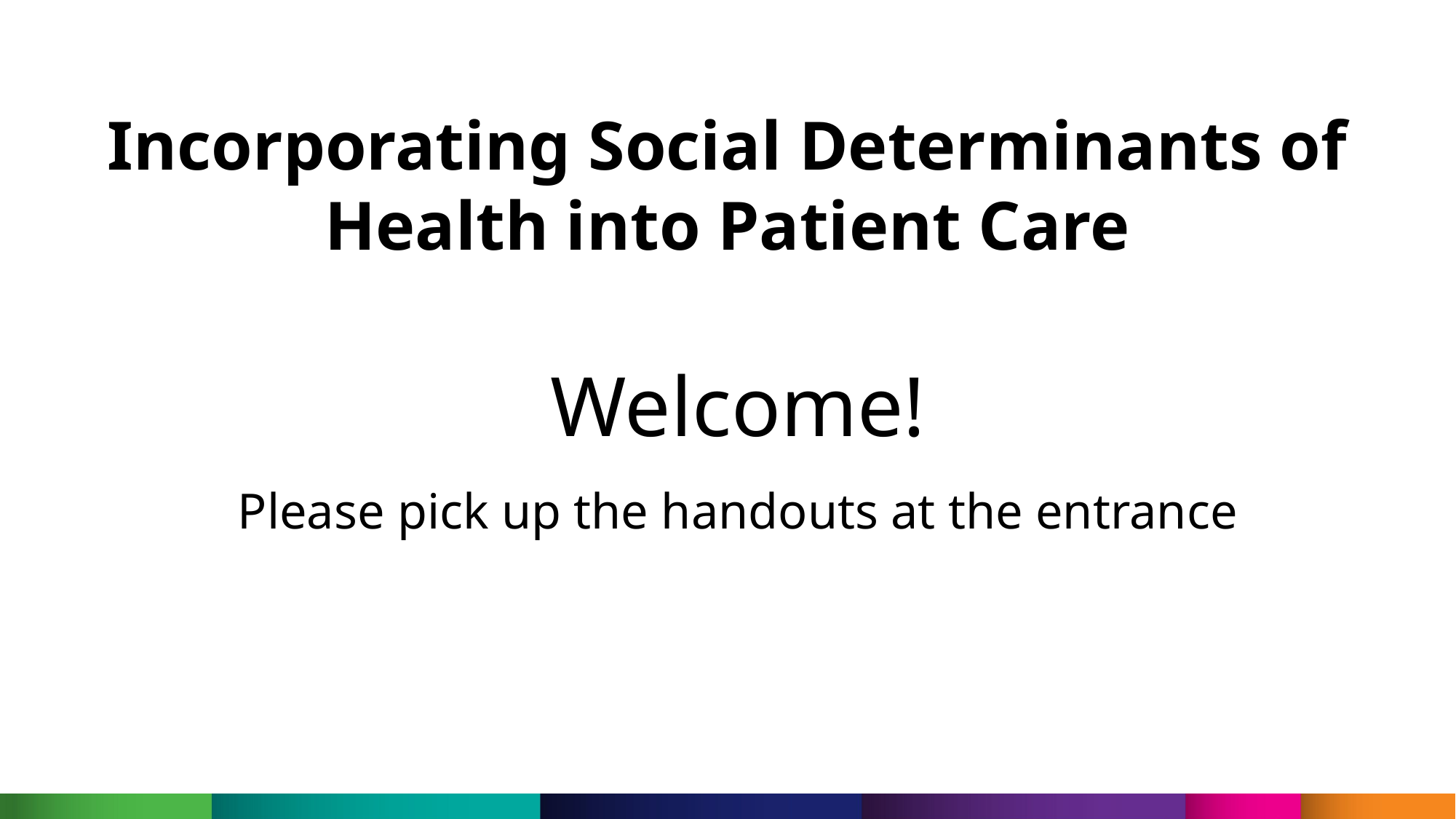

# Incorporating Social Determinants of Health into Patient Care
Welcome!
Please pick up the handouts at the entrance

## Slide 2
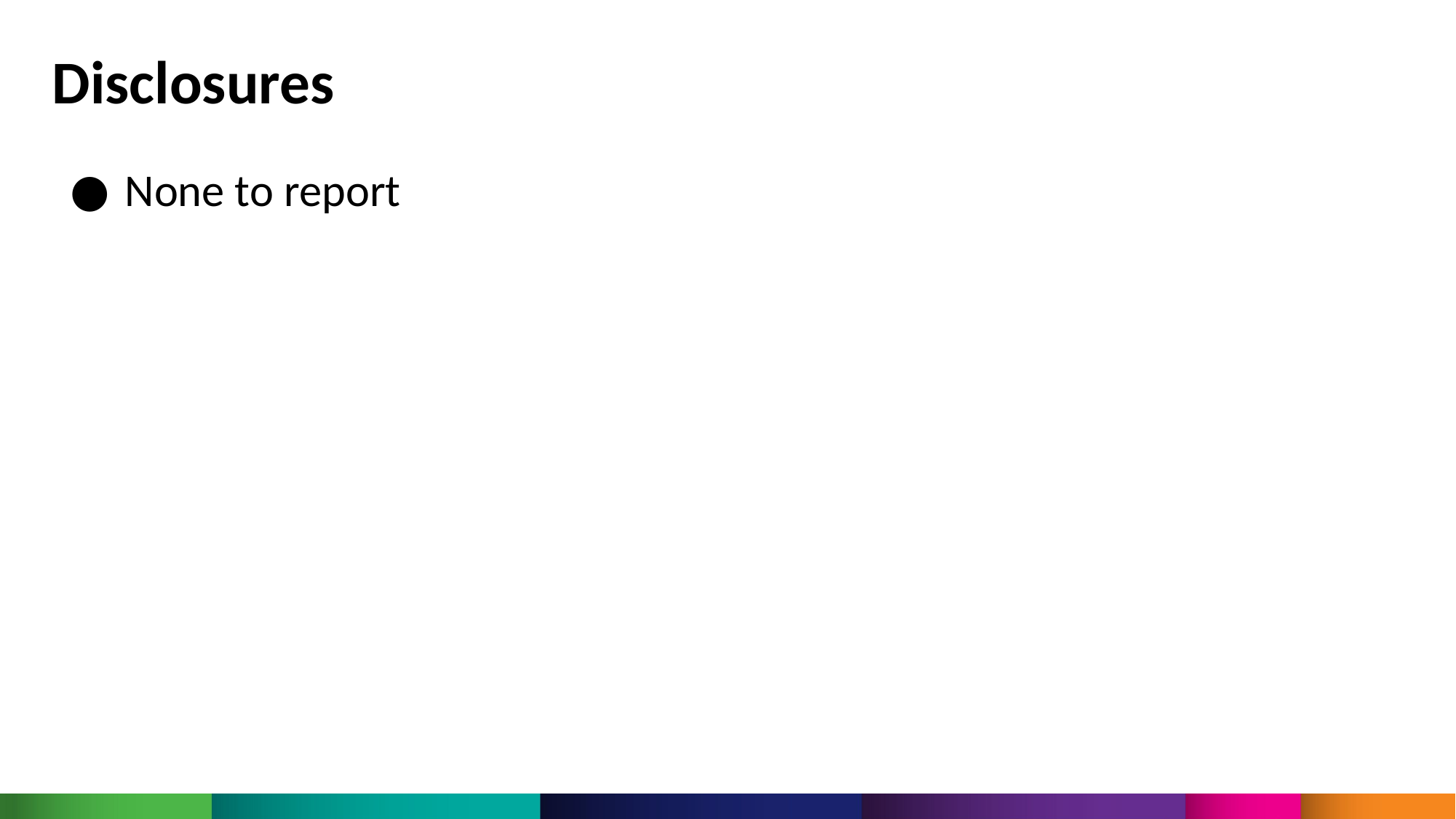

# Disclosures
None to report

## Slide 3
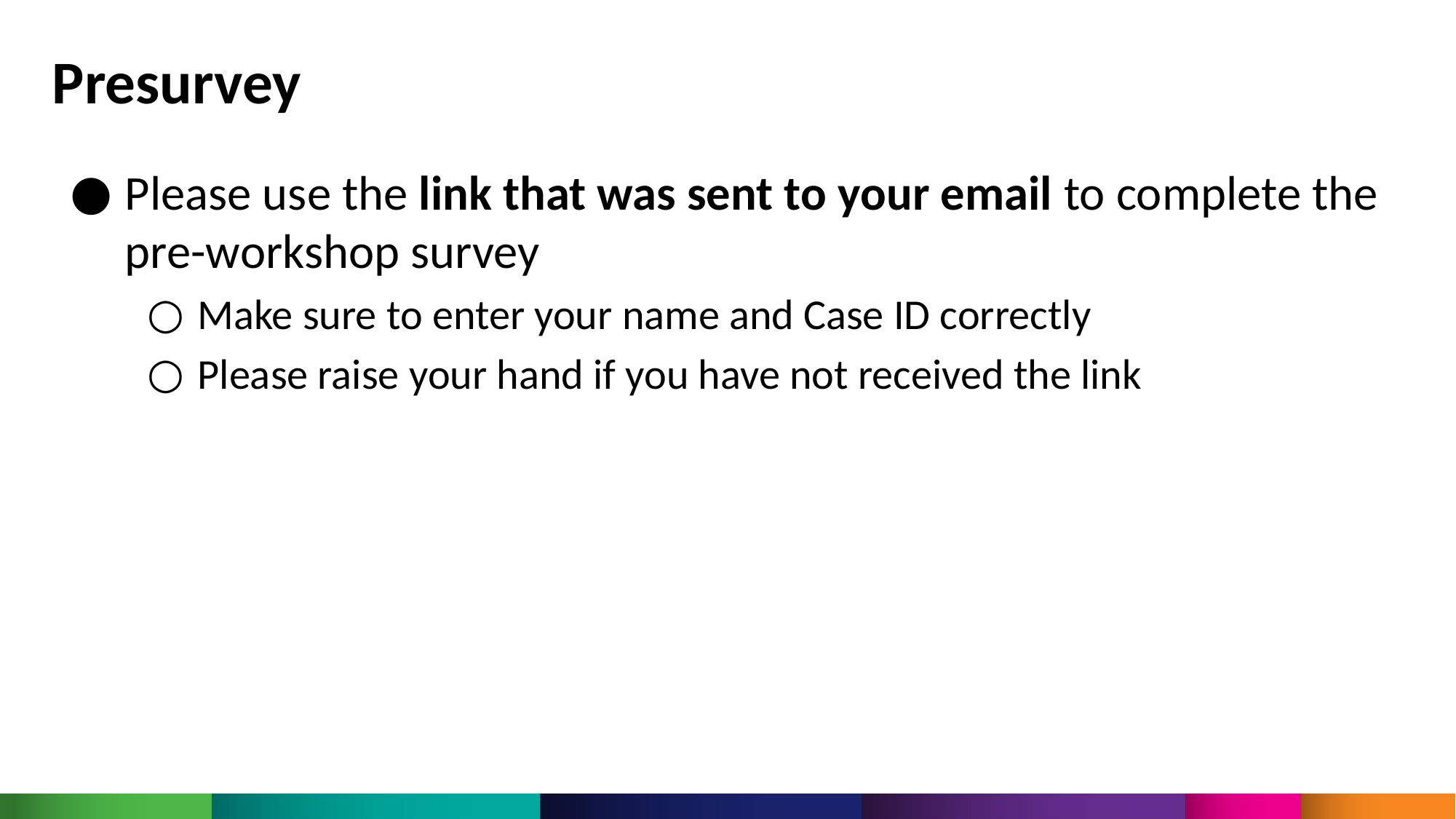

# Presurvey
Please use the link that was sent to your email to complete the pre-workshop survey
Make sure to enter your name and Case ID correctly
Please raise your hand if you have not received the link

## Slide 4
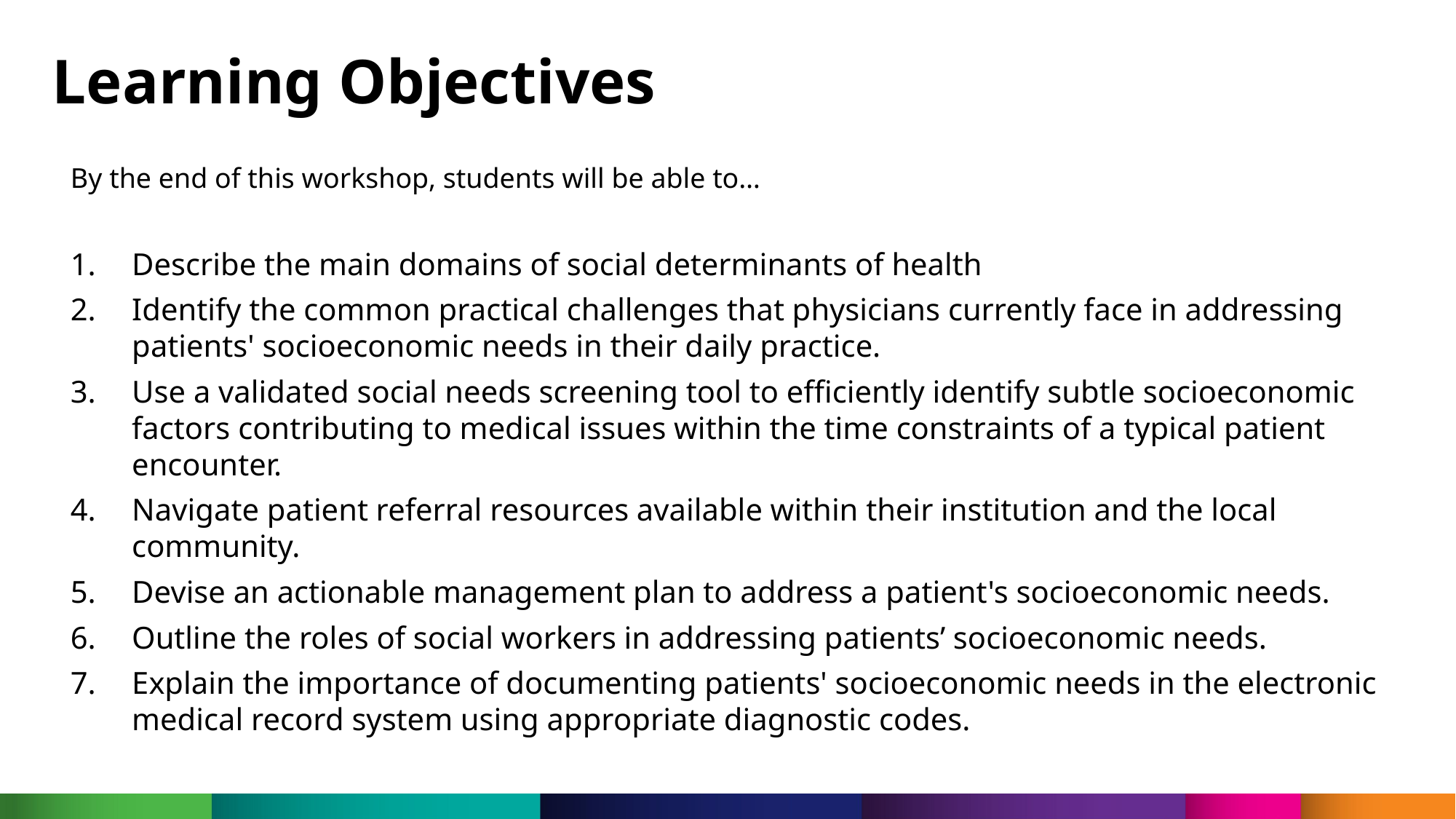

# Learning Objectives
By the end of this workshop, students will be able to…
Describe the main domains of social determinants of health
Identify the common practical challenges that physicians currently face in addressing patients' socioeconomic needs in their daily practice.
Use a validated social needs screening tool to efficiently identify subtle socioeconomic factors contributing to medical issues within the time constraints of a typical patient encounter.
Navigate patient referral resources available within their institution and the local community.
Devise an actionable management plan to address a patient's socioeconomic needs.
Outline the roles of social workers in addressing patients’ socioeconomic needs.
Explain the importance of documenting patients' socioeconomic needs in the electronic medical record system using appropriate diagnostic codes.

## Slide 5
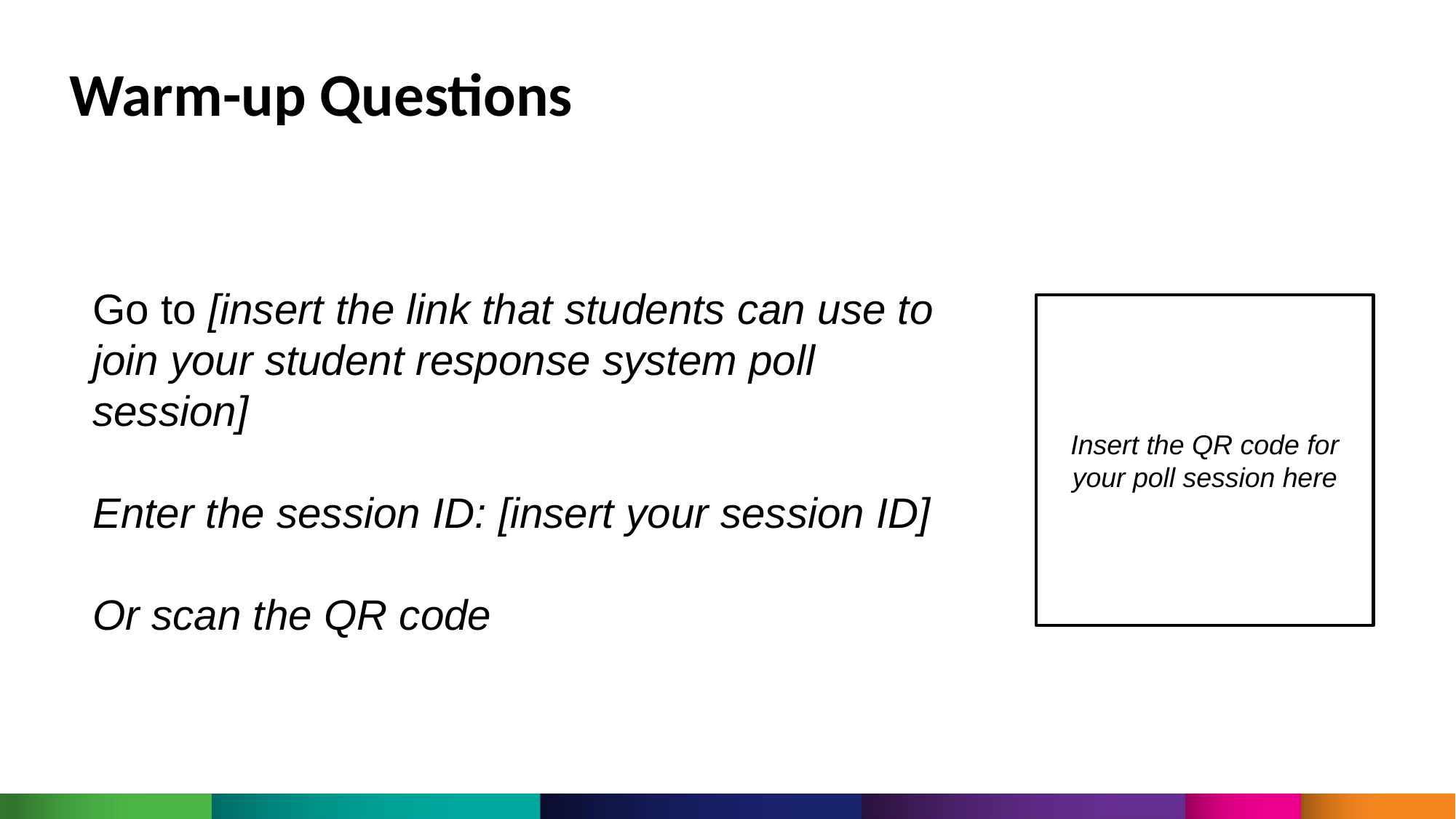

Warm-up Questions
Go to [insert the link that students can use to join your student response system poll session]
Enter the session ID: [insert your session ID]
Or scan the QR code
Insert the QR code for your poll session here

## Slide 6
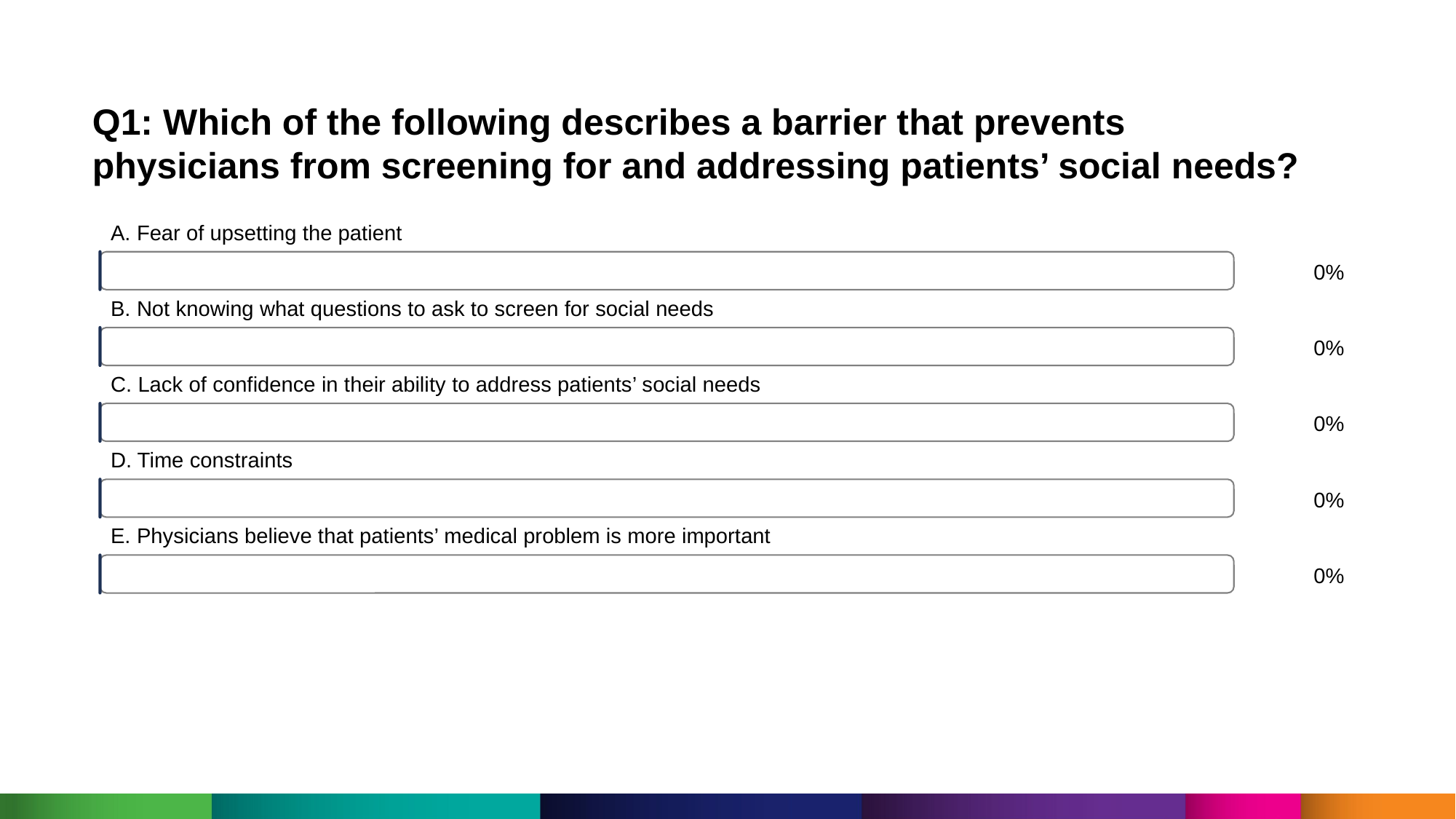

Q1: Which of the following describes a barrier that prevents physicians from screening for and addressing patients’ social needs?
A. Fear of upsetting the patient
0%
B. Not knowing what questions to ask to screen for social needs
0%
C. Lack of confidence in their ability to address patients’ social needs
0%
D. Time constraints
0%
E. Physicians believe that patients’ medical problem is more important
0%

## Slide 7
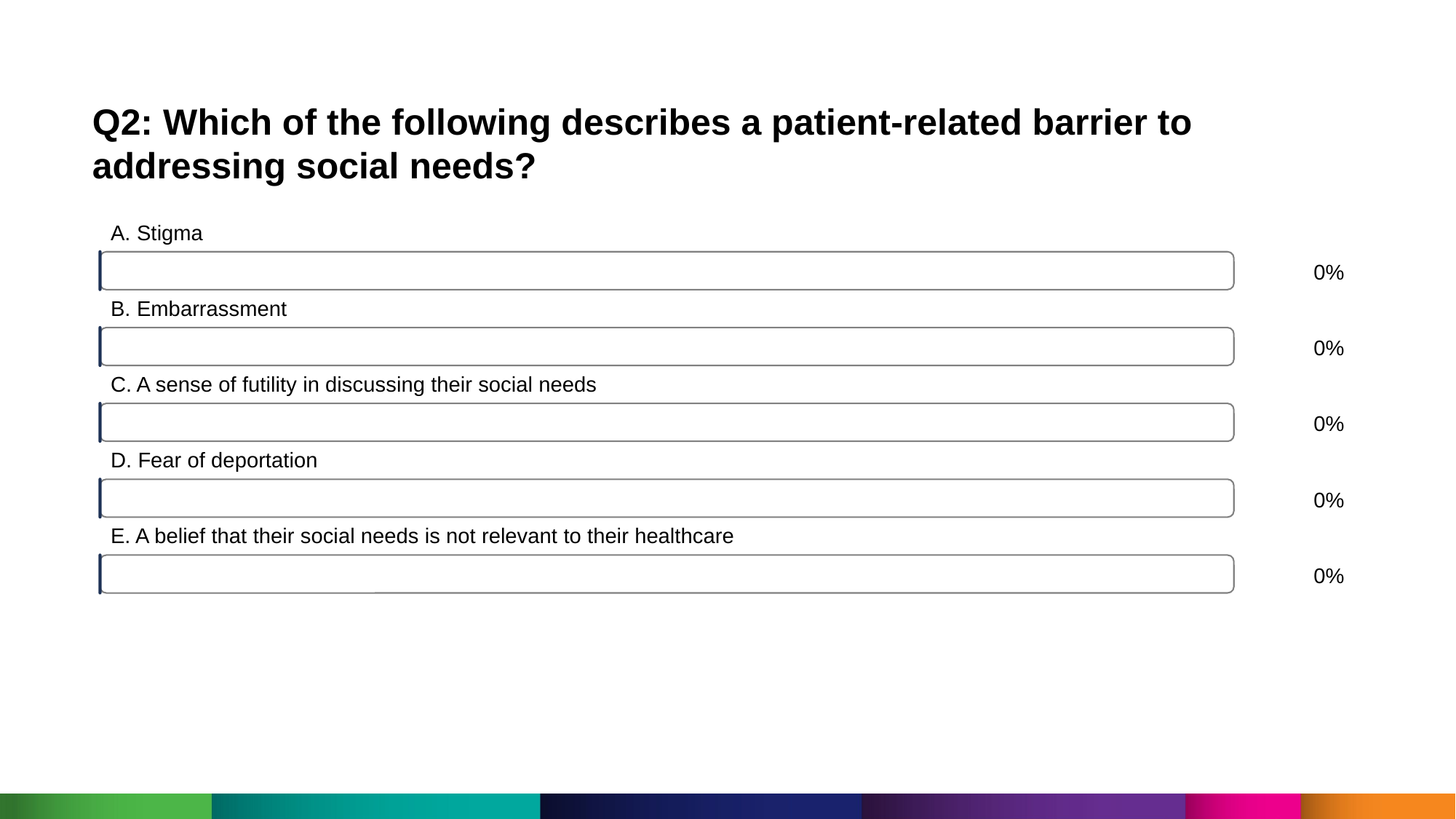

Q2: Which of the following describes a patient-related barrier to addressing social needs?
A. Stigma
0%
B. Embarrassment
0%
C. A sense of futility in discussing their social needs
0%
D. Fear of deportation
0%
E. A belief that their social needs is not relevant to their healthcare
0%

## Slide 8
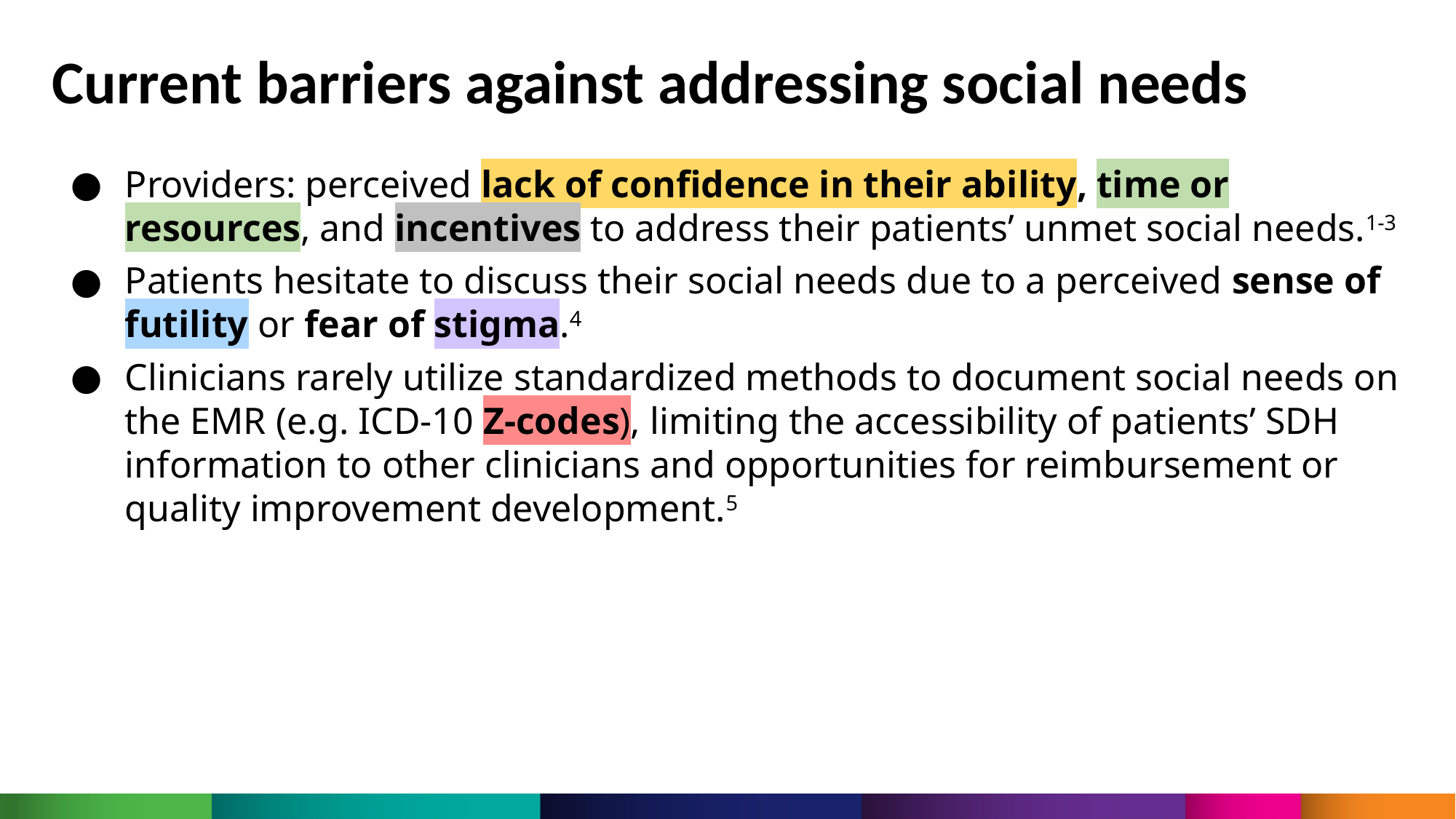

# Current barriers against addressing social needs
Providers: perceived lack of confidence in their ability, time or resources, and incentives to address their patients’ unmet social needs.1-3
Patients hesitate to discuss their social needs due to a perceived sense of futility or fear of stigma.4
Clinicians rarely utilize standardized methods to document social needs on the EMR (e.g. ICD-10 Z-codes), limiting the accessibility of patients’ SDH information to other clinicians and opportunities for reimbursement or quality improvement development.5

## Slide 9
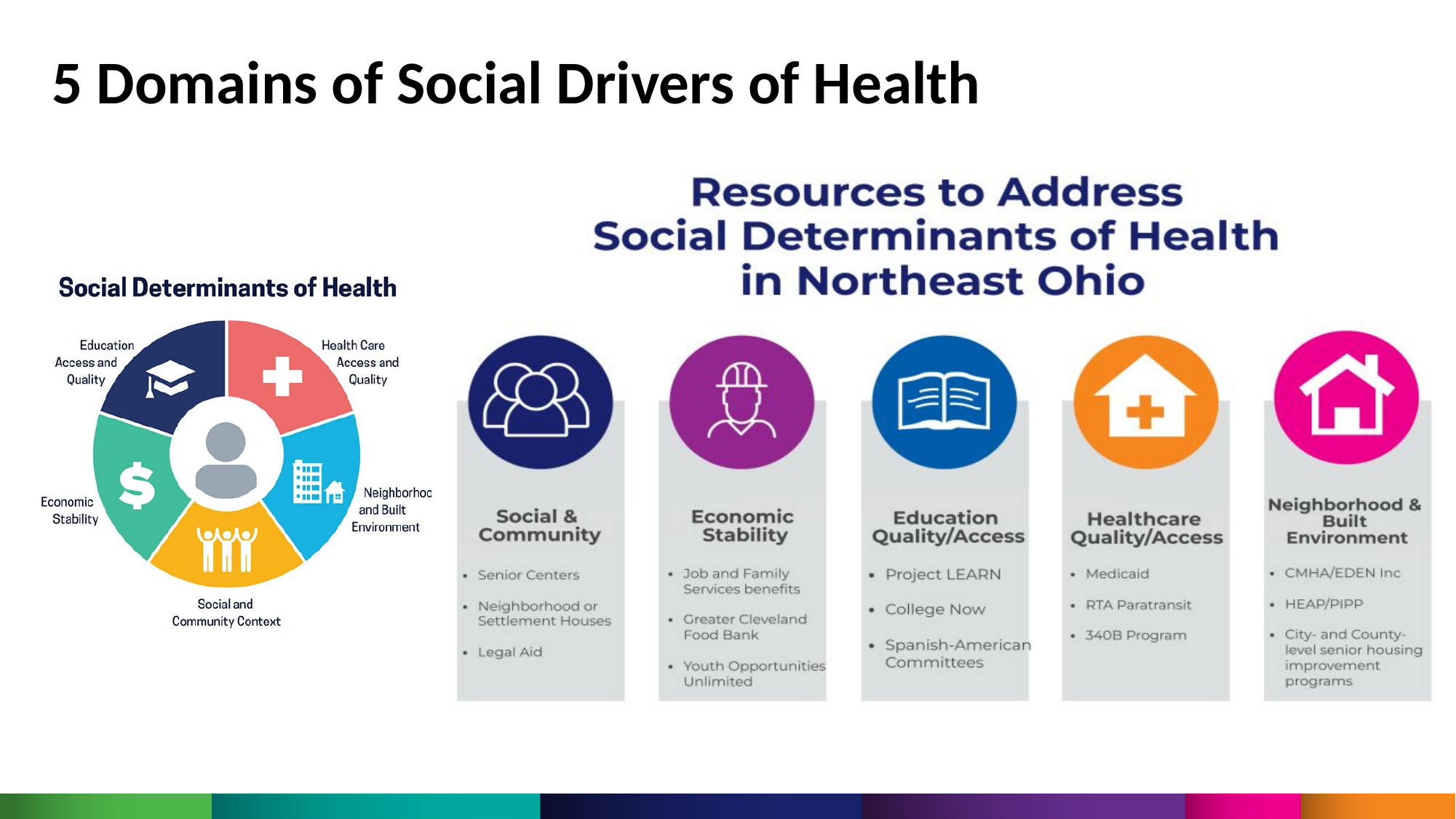

# 5 Domains of Social Drivers of Health

## Slide 10
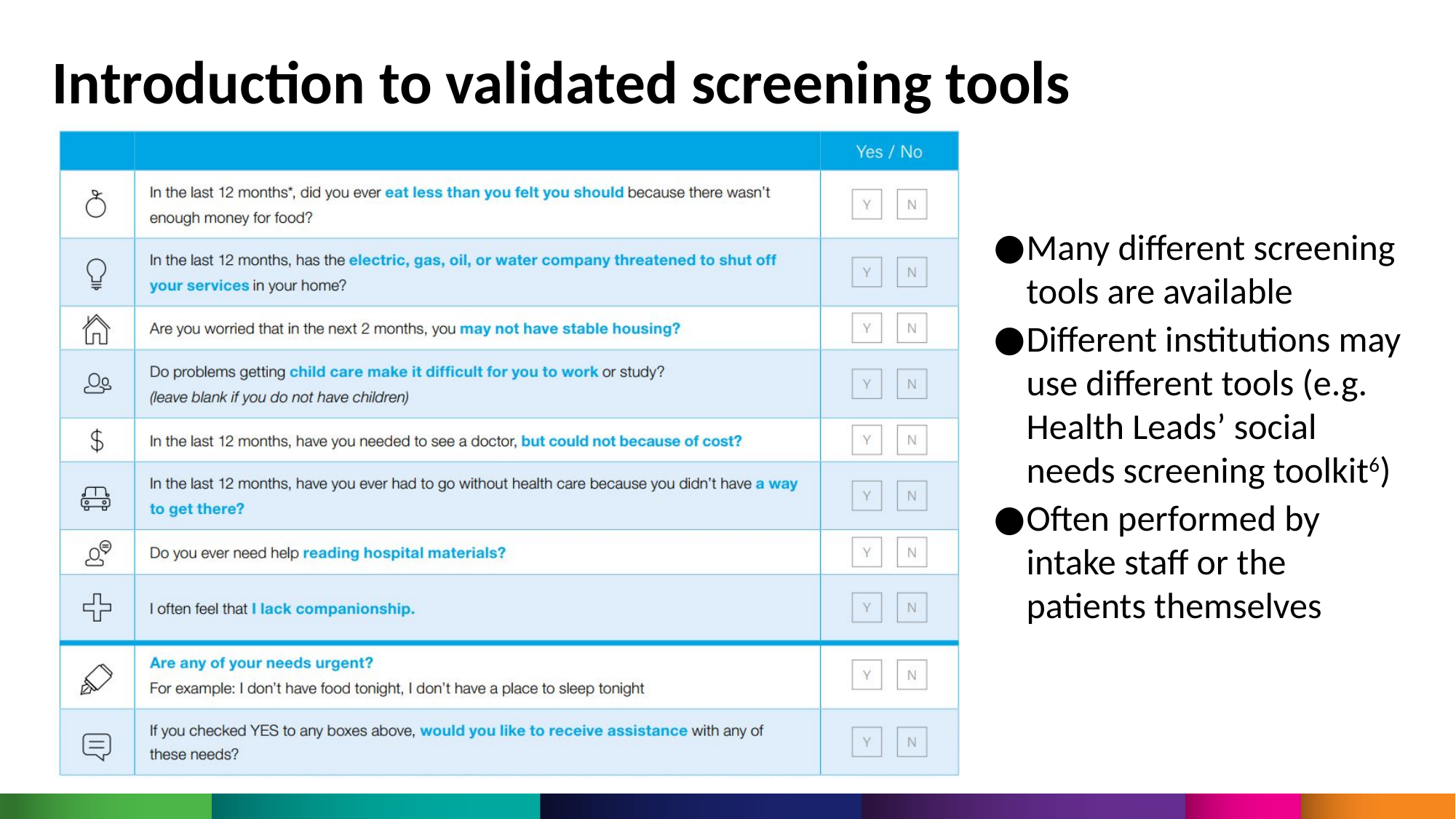

# Introduction to validated screening tools
Many different screening tools are available
Different institutions may use different tools (e.g. Health Leads’ social needs screening toolkit6)
Often performed by intake staff or the patients themselves

## Slide 11
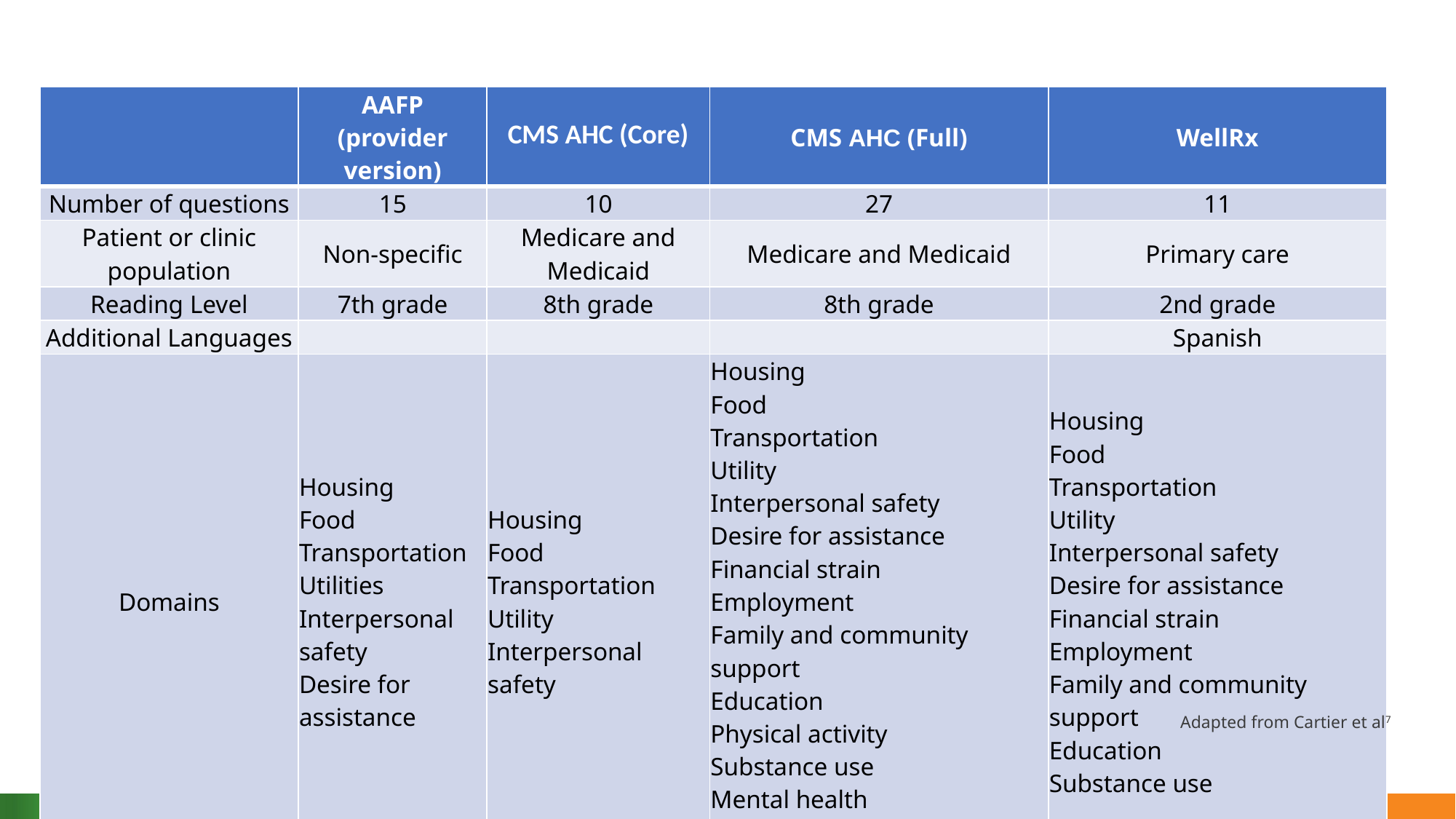

| | AAFP (provider version) | CMS AHC (Core) | CMS AHC (Full) | WellRx |
| --- | --- | --- | --- | --- |
| Number of questions | 15 | 10 | 27 | 11 |
| Patient or clinic population | Non-specific | Medicare and Medicaid | Medicare and Medicaid | Primary care |
| Reading Level | 7th grade | 8th grade | 8th grade | 2nd grade |
| Additional Languages | | | | Spanish |
| Domains | Housing Food Transportation Utilities Interpersonal safety Desire for assistance | Housing Food Transportation Utility Interpersonal safety | Housing Food Transportation Utility Interpersonal safety Desire for assistance Financial strain Employment Family and community support Education Physical activity Substance use Mental health Disabilities | Housing Food Transportation Utility Interpersonal safety Desire for assistance Financial strain Employment Family and community support Education Substance use |
Adapted from Cartier et al7

## Slide 12
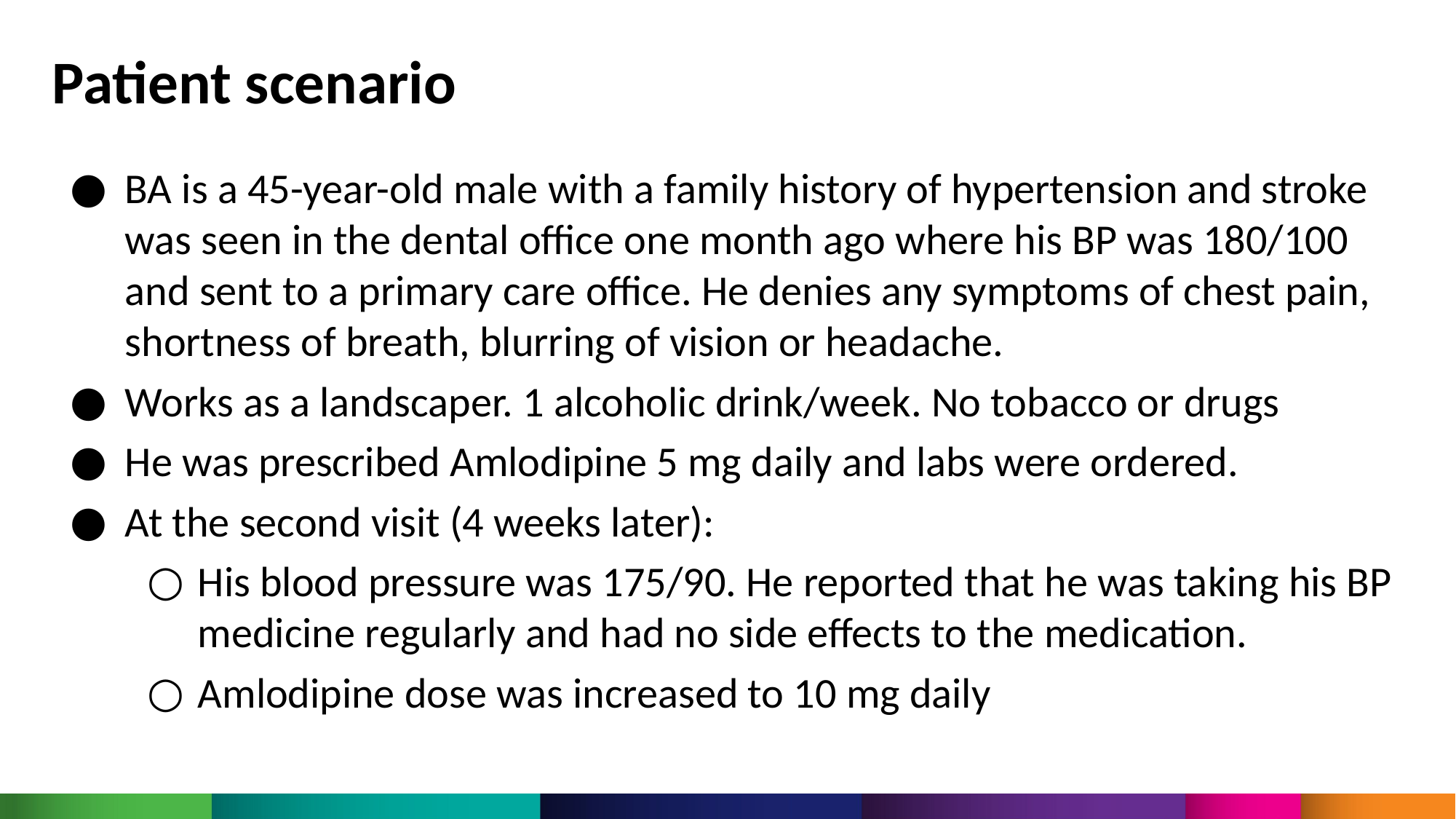

# Patient scenario
BA is a 45-year-old male with a family history of hypertension and stroke was seen in the dental office one month ago where his BP was 180/100 and sent to a primary care office. He denies any symptoms of chest pain, shortness of breath, blurring of vision or headache.
Works as a landscaper. 1 alcoholic drink/week. No tobacco or drugs
He was prescribed Amlodipine 5 mg daily and labs were ordered.
At the second visit (4 weeks later):
His blood pressure was 175/90. He reported that he was taking his BP medicine regularly and had no side effects to the medication.
Amlodipine dose was increased to 10 mg daily

## Slide 13
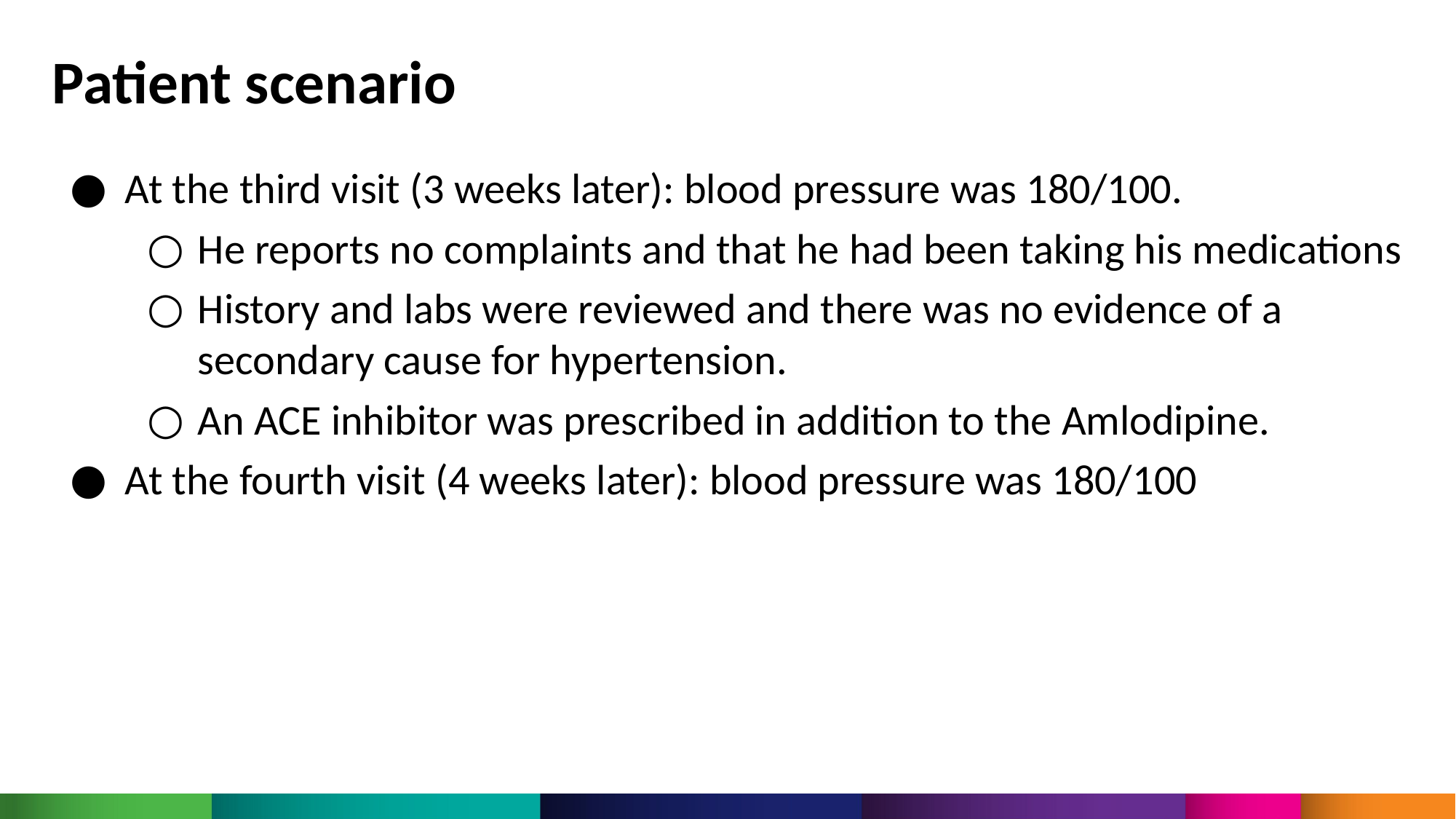

# Patient scenario
At the third visit (3 weeks later): blood pressure was 180/100.
He reports no complaints and that he had been taking his medications
History and labs were reviewed and there was no evidence of a secondary cause for hypertension.
An ACE inhibitor was prescribed in addition to the Amlodipine.
At the fourth visit (4 weeks later): blood pressure was 180/100

## Slide 14
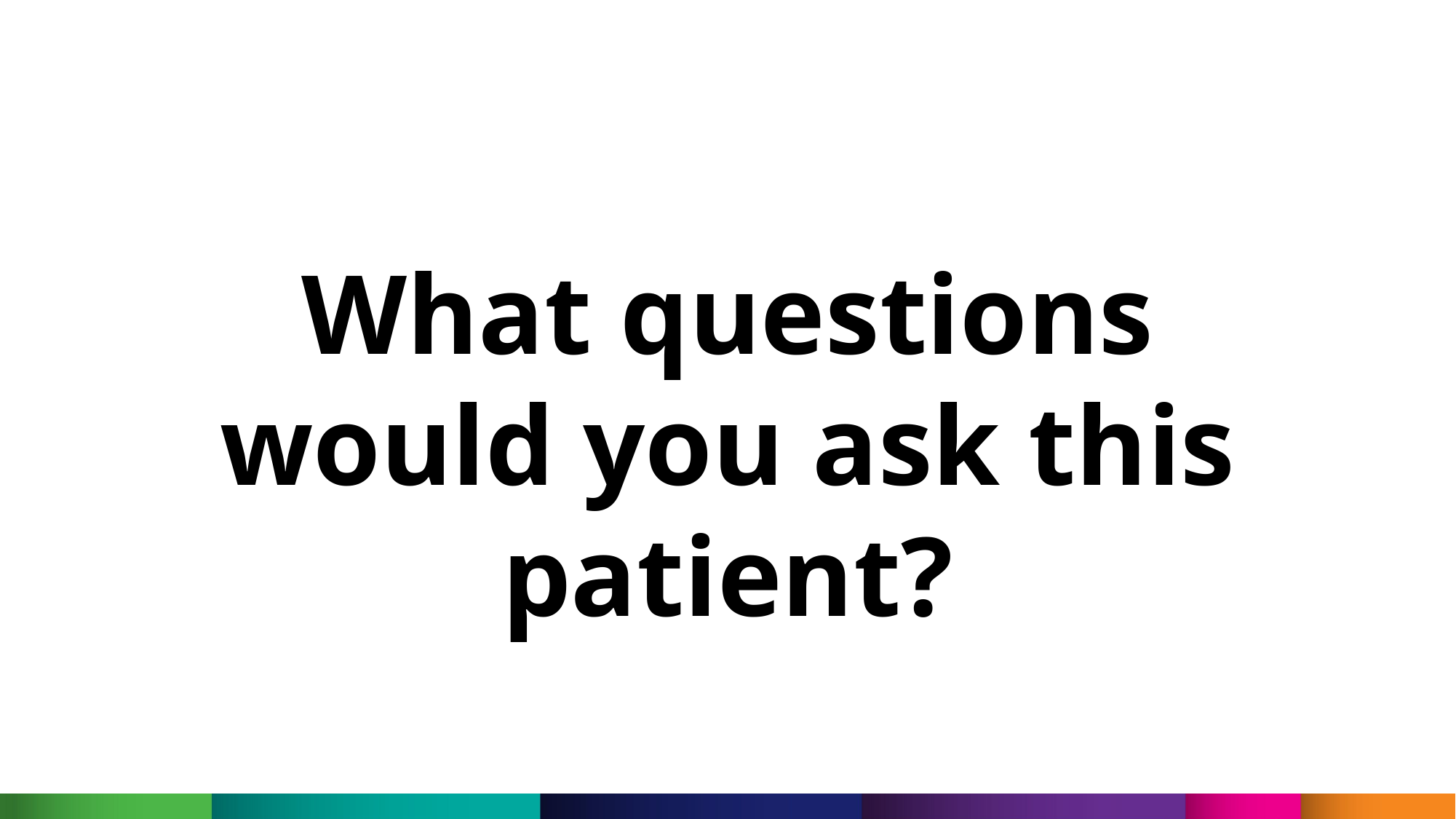

# What questions would you ask this patient?

## Slide 15
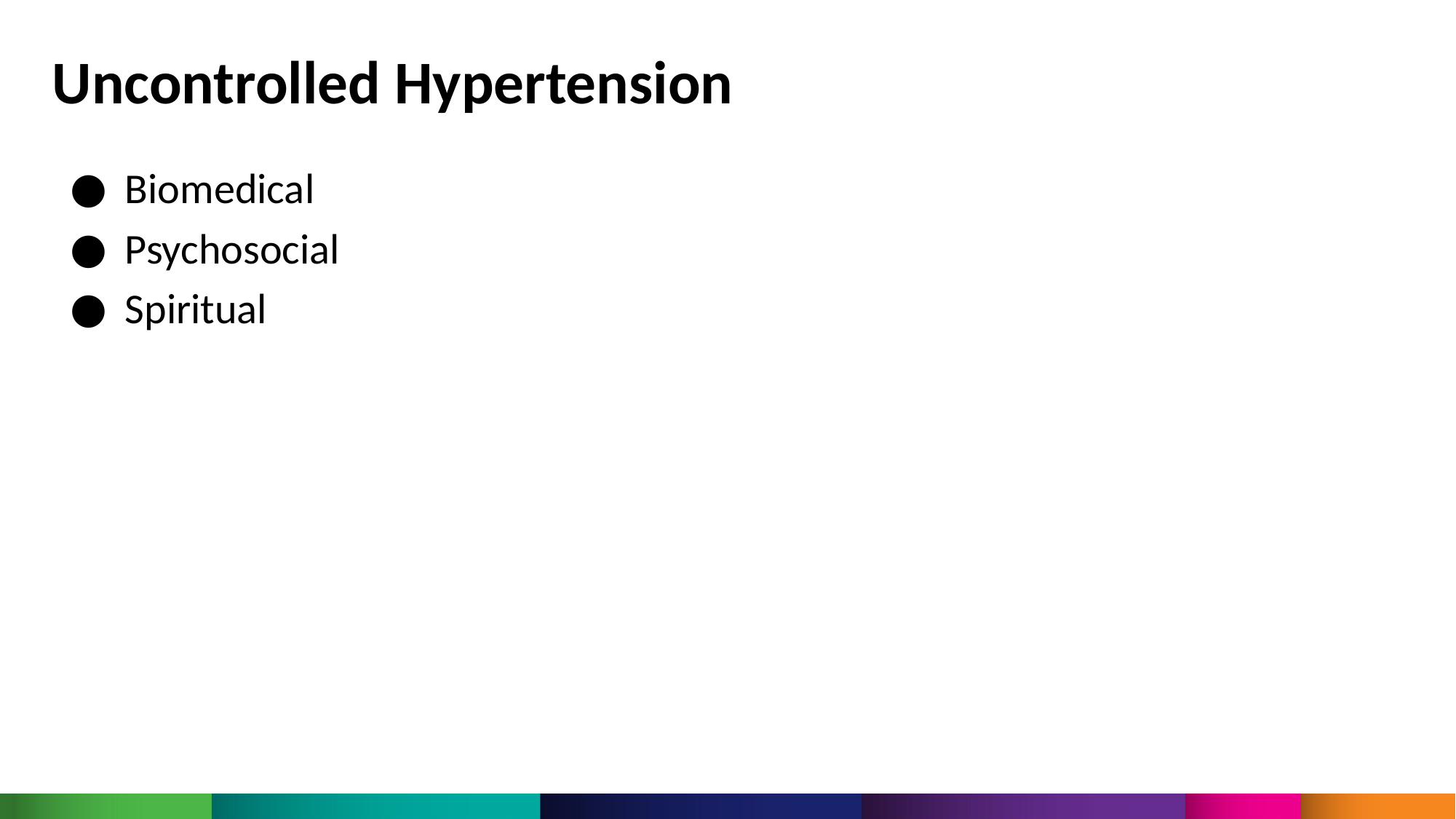

# Uncontrolled Hypertension
Biomedical
Psychosocial
Spiritual

## Slide 16
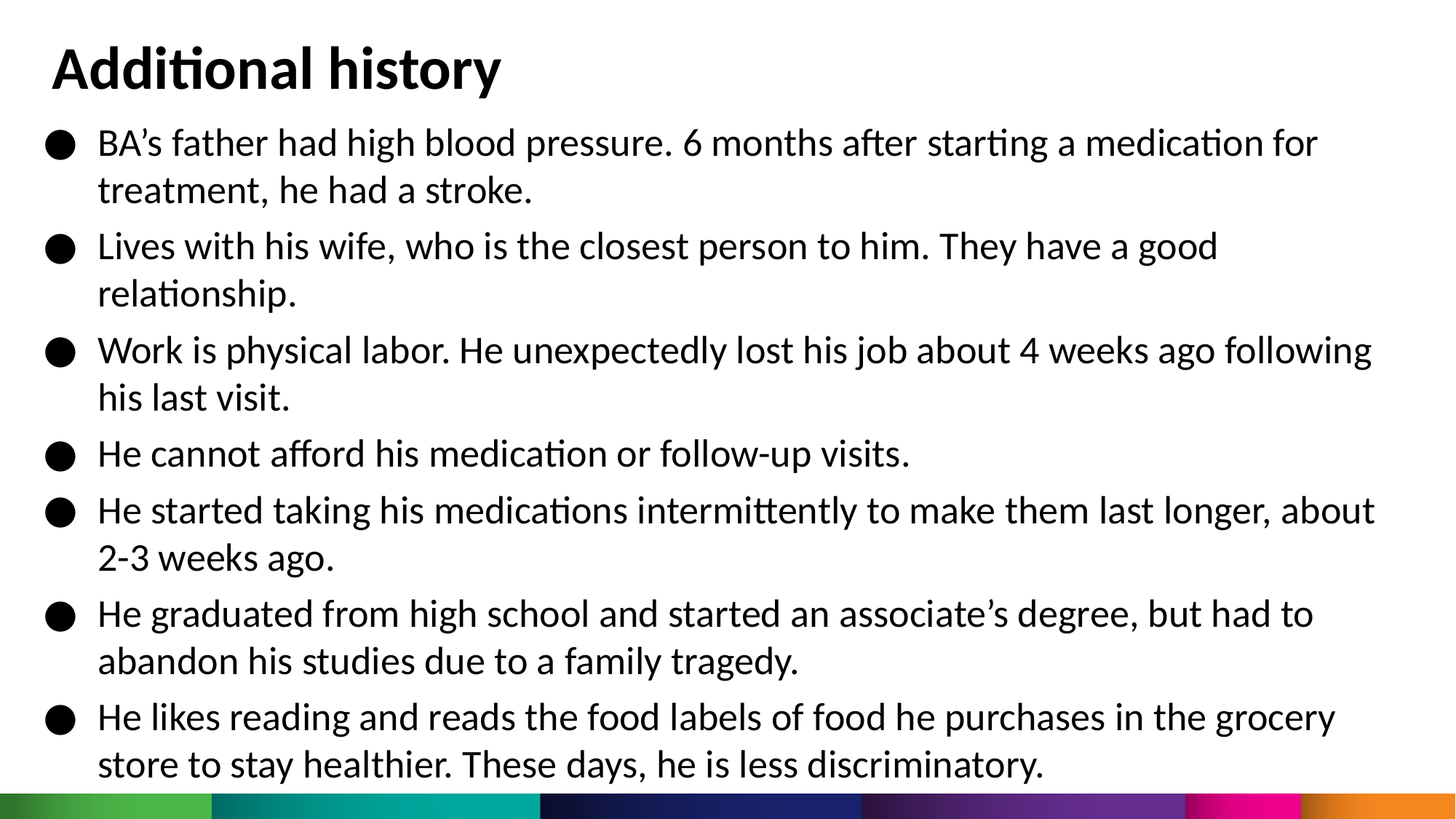

# Additional history
BA’s father had high blood pressure. 6 months after starting a medication for treatment, he had a stroke.
Lives with his wife, who is the closest person to him. They have a good relationship.
Work is physical labor. He unexpectedly lost his job about 4 weeks ago following his last visit.
He cannot afford his medication or follow-up visits.
He started taking his medications intermittently to make them last longer, about 2-3 weeks ago.
He graduated from high school and started an associate’s degree, but had to abandon his studies due to a family tragedy.
He likes reading and reads the food labels of food he purchases in the grocery store to stay healthier. These days, he is less discriminatory.

## Slide 17
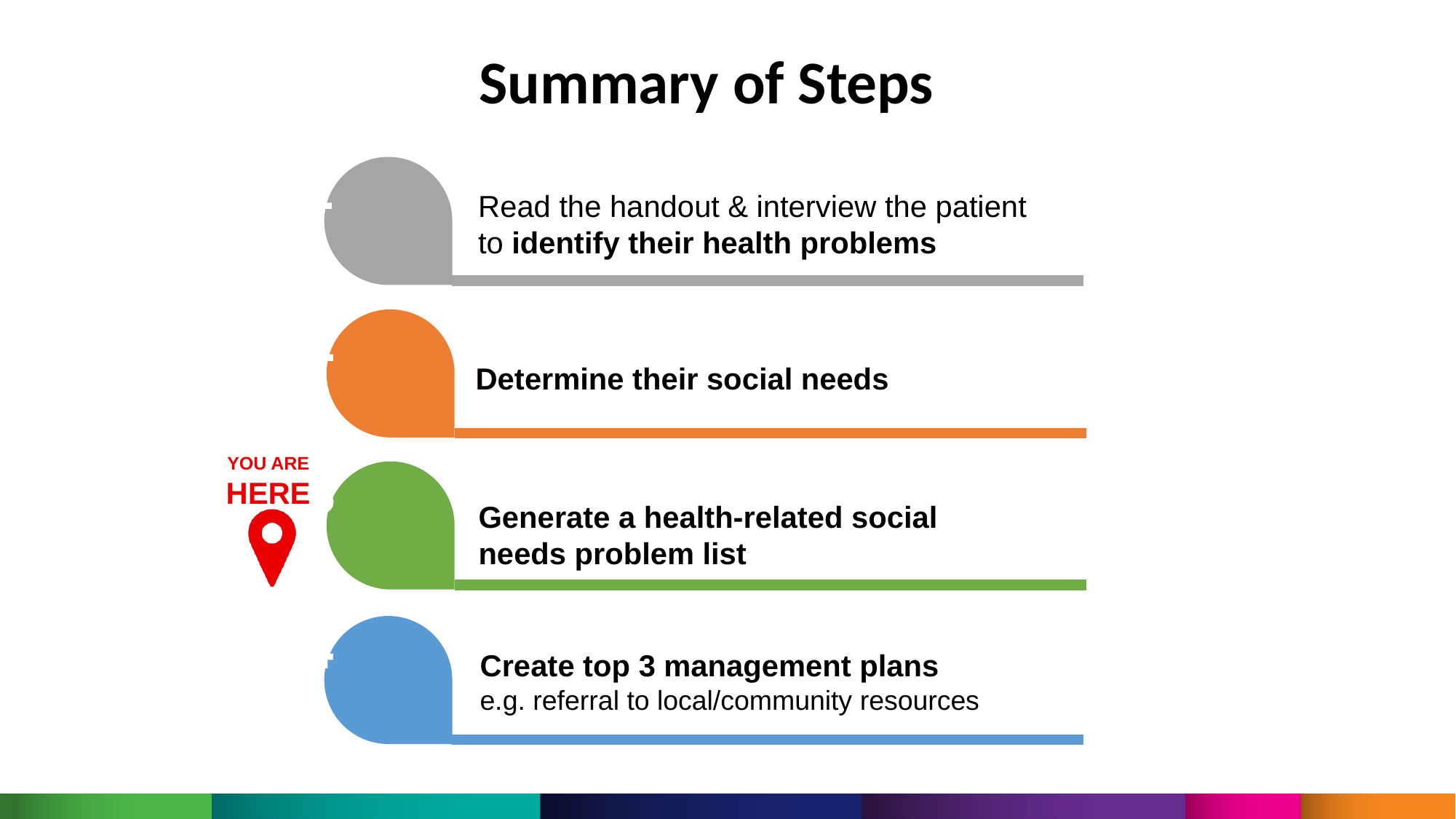

Summary of Steps
1
Read the handout & interview the patient to identify their health problems
2
Determine their social needs
3
Generate a health-related social needs problem list
4
Create top 3 management plans
e.g. referral to local/community resources
YOU ARE
HERE

## Slide 18
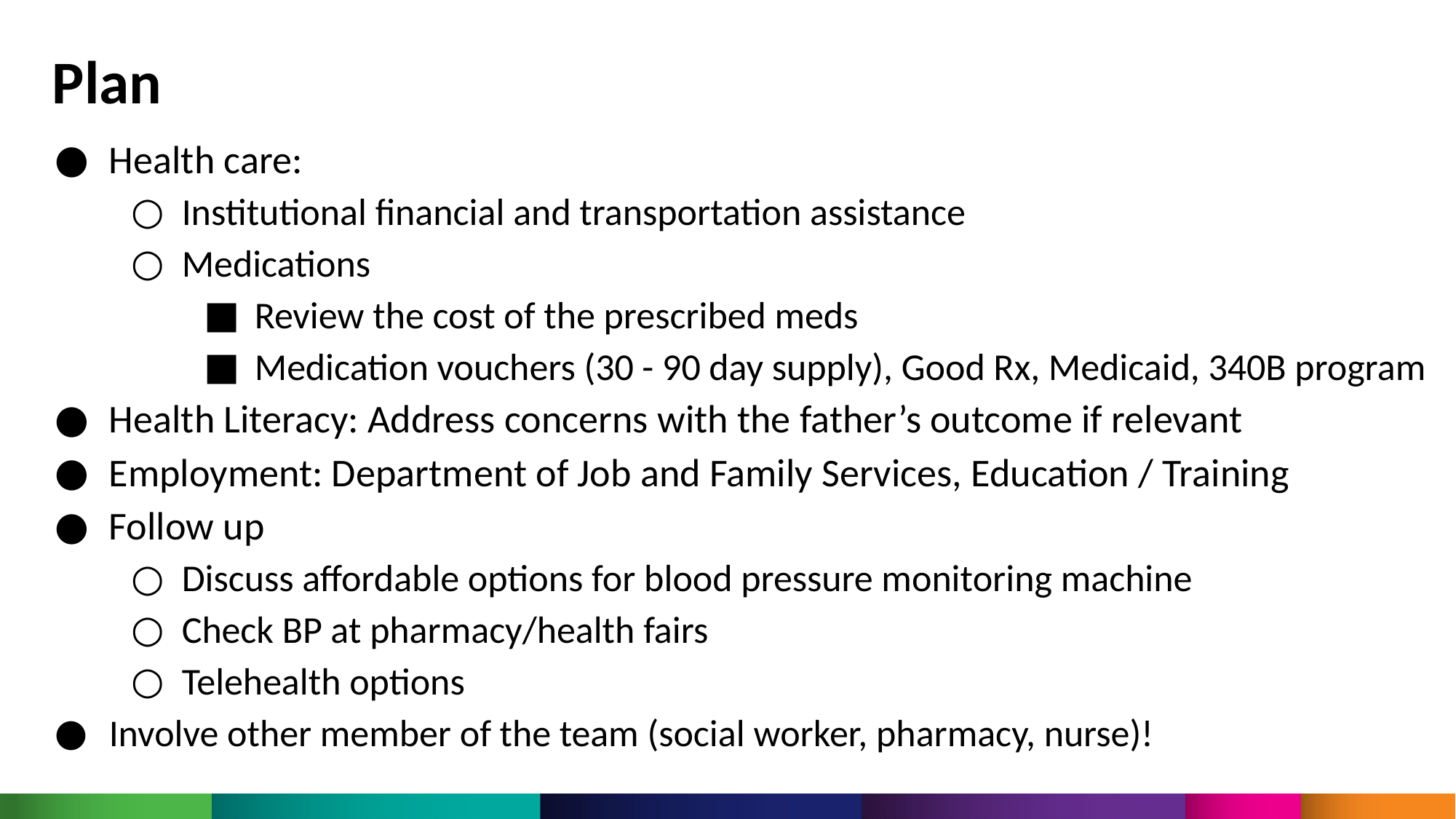

# Plan
Health care:
Institutional financial and transportation assistance
Medications
Review the cost of the prescribed meds
Medication vouchers (30 - 90 day supply), Good Rx, Medicaid, 340B program
Health Literacy: Address concerns with the father’s outcome if relevant
Employment: Department of Job and Family Services, Education / Training
Follow up
Discuss affordable options for blood pressure monitoring machine
Check BP at pharmacy/health fairs
Telehealth options
Involve other member of the team (social worker, pharmacy, nurse)!

## Slide 19
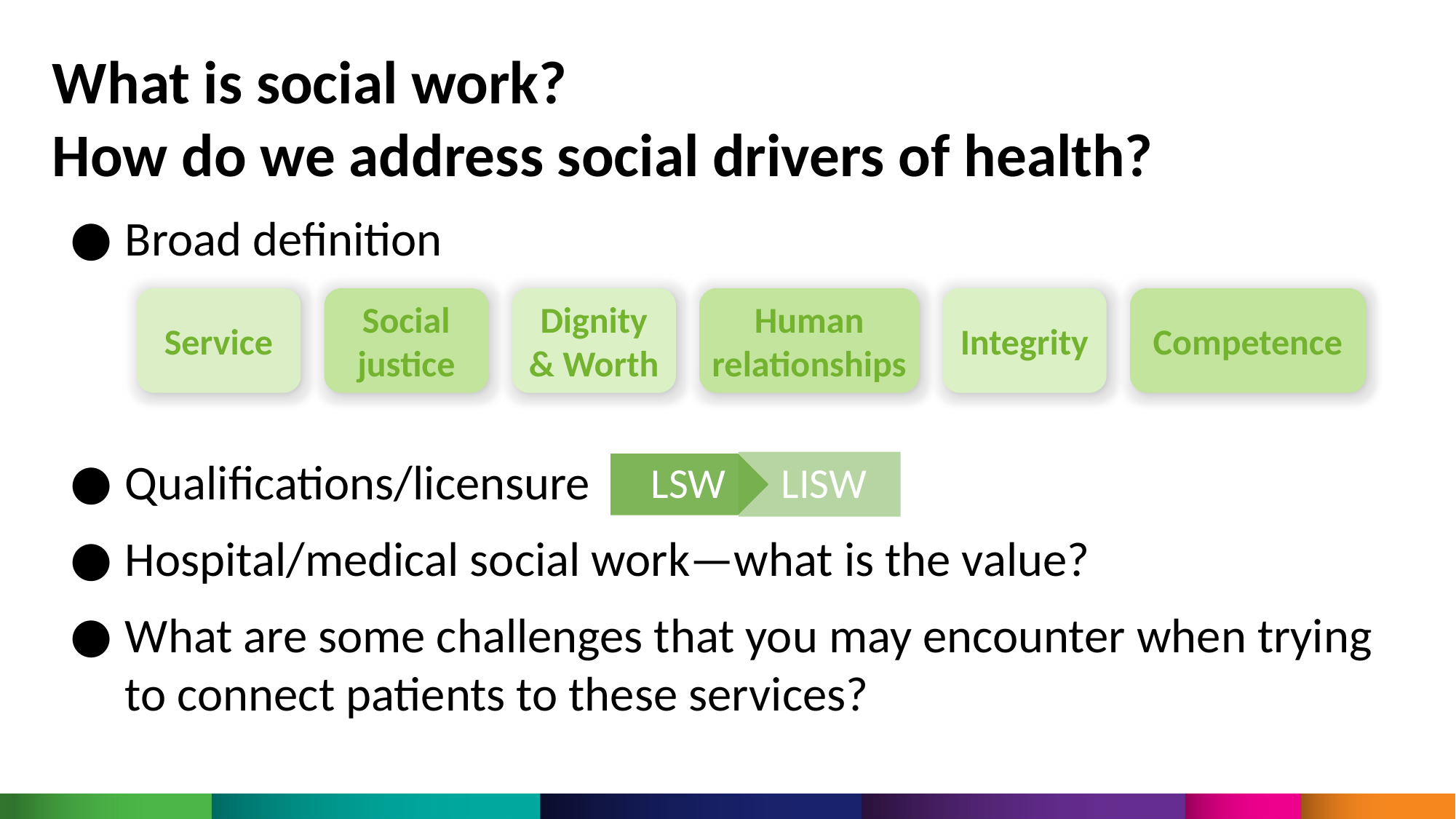

What is social work?
How do we address social drivers of health?
Broad definition
Qualifications/licensure
Hospital/medical social work—what is the value?
What are some challenges that you may encounter when trying to connect patients to these services?
Service
Social justice
Dignity
& Worth
Human relationships
Integrity
Competence

## Slide 20
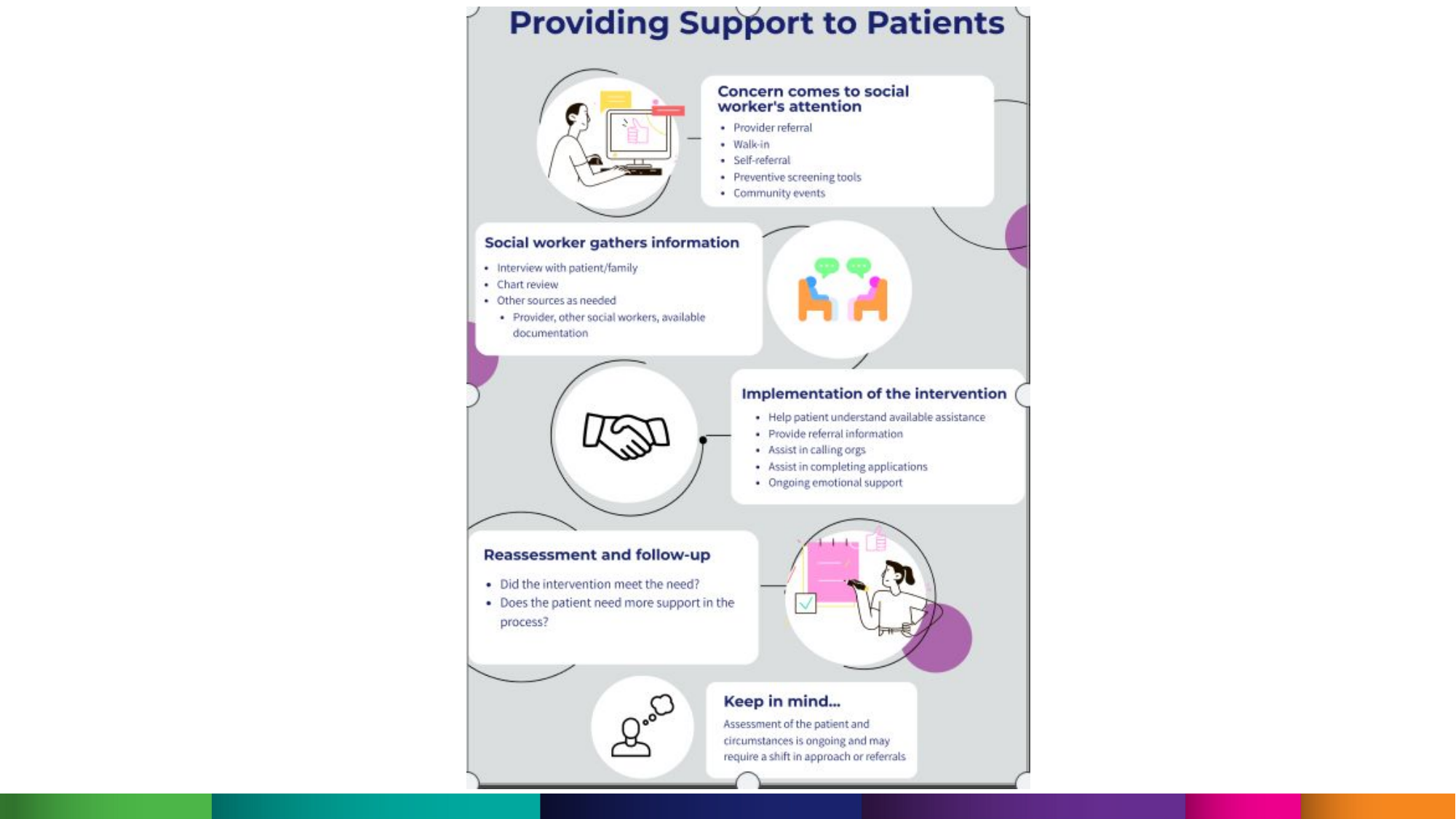

## Slide 21
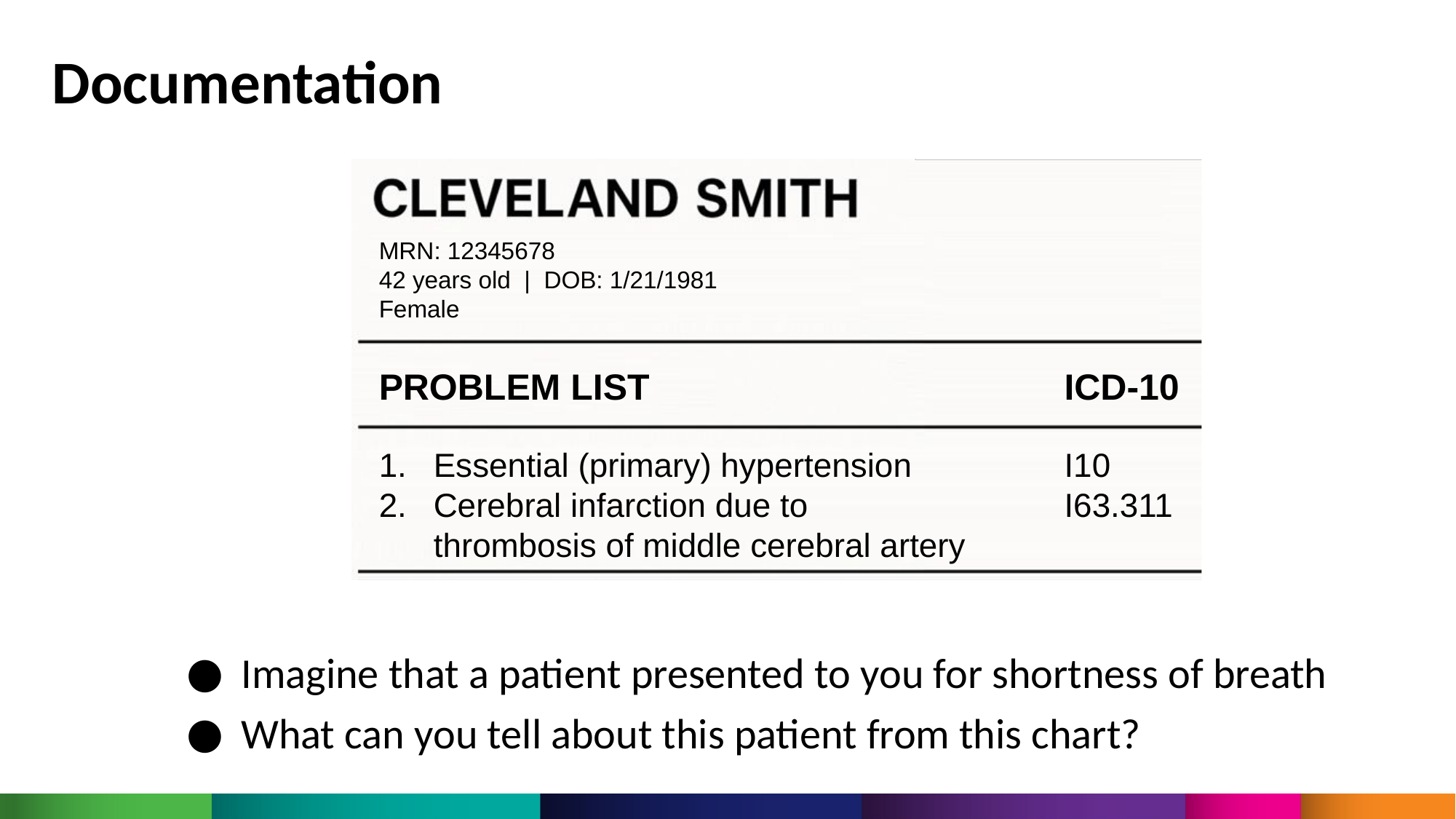

# Documentation
MRN: 12345678
42 years old | DOB: 1/21/1981
Female
PROBLEM LIST
ICD-10
Essential (primary) hypertension
Cerebral infarction due to thrombosis of middle cerebral artery
I10
I63.311
Imagine that a patient presented to you for shortness of breath
What can you tell about this patient from this chart?

## Slide 22
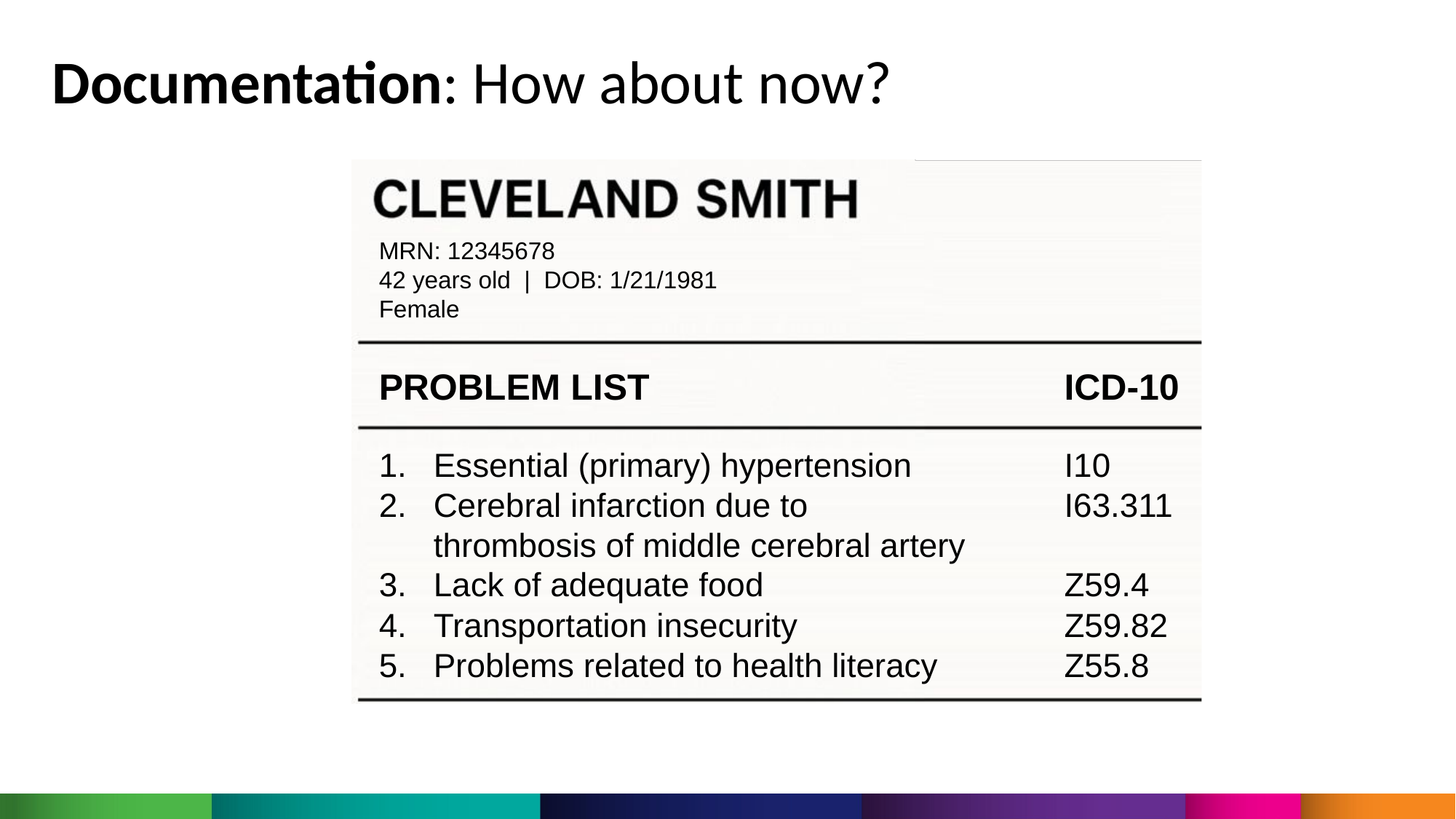

# Documentation: How about now?
MRN: 12345678
42 years old | DOB: 1/21/1981
Female
PROBLEM LIST
ICD-10
Essential (primary) hypertension
Cerebral infarction due to thrombosis of middle cerebral artery
Lack of adequate food
Transportation insecurity
Problems related to health literacy
I10
I63.311
Z59.4
Z59.82
Z55.8

## Slide 23
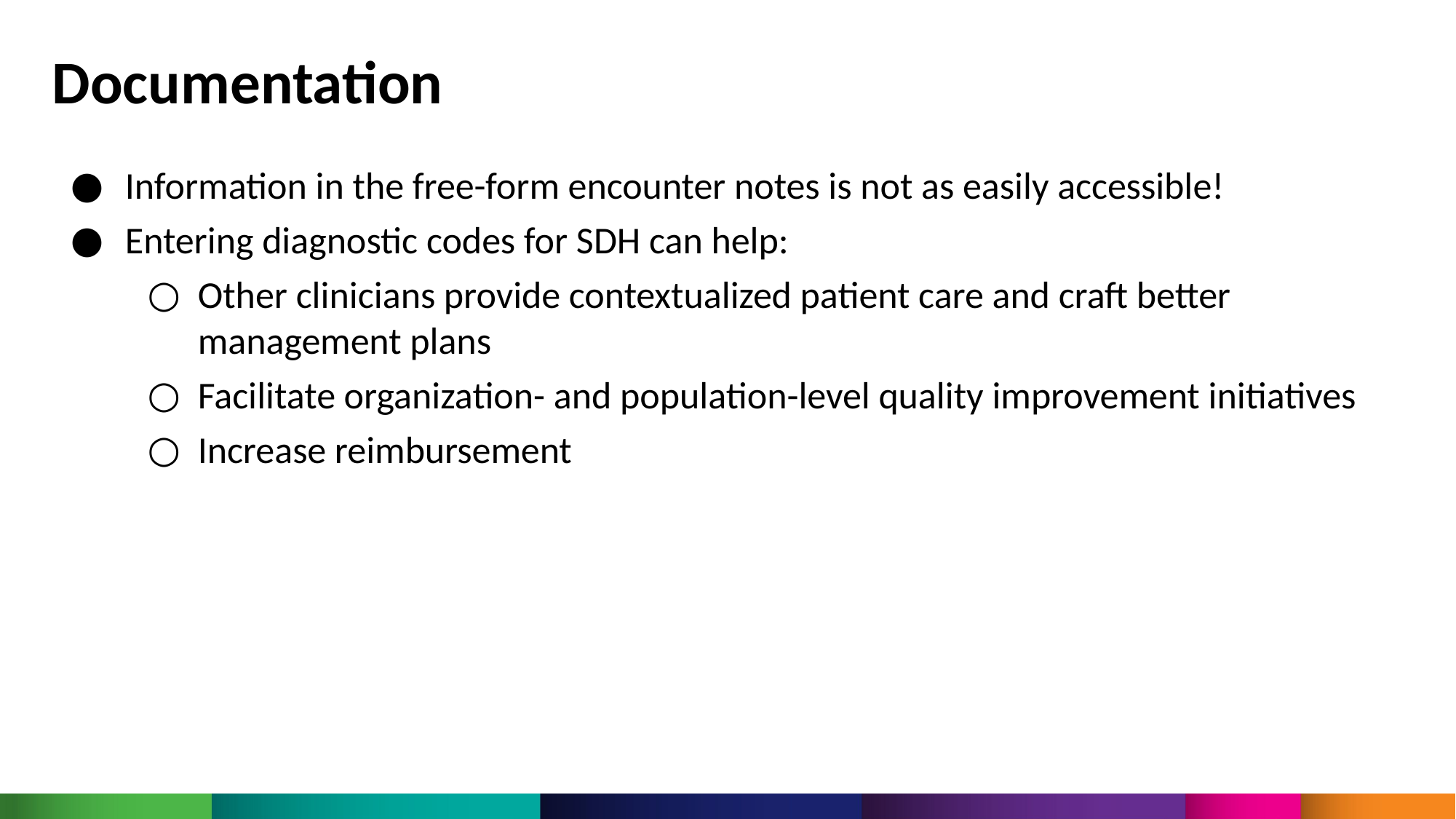

# Documentation
Information in the free-form encounter notes is not as easily accessible!
Entering diagnostic codes for SDH can help:
Other clinicians provide contextualized patient care and craft better management plans
Facilitate organization- and population-level quality improvement initiatives
Increase reimbursement

## Slide 24
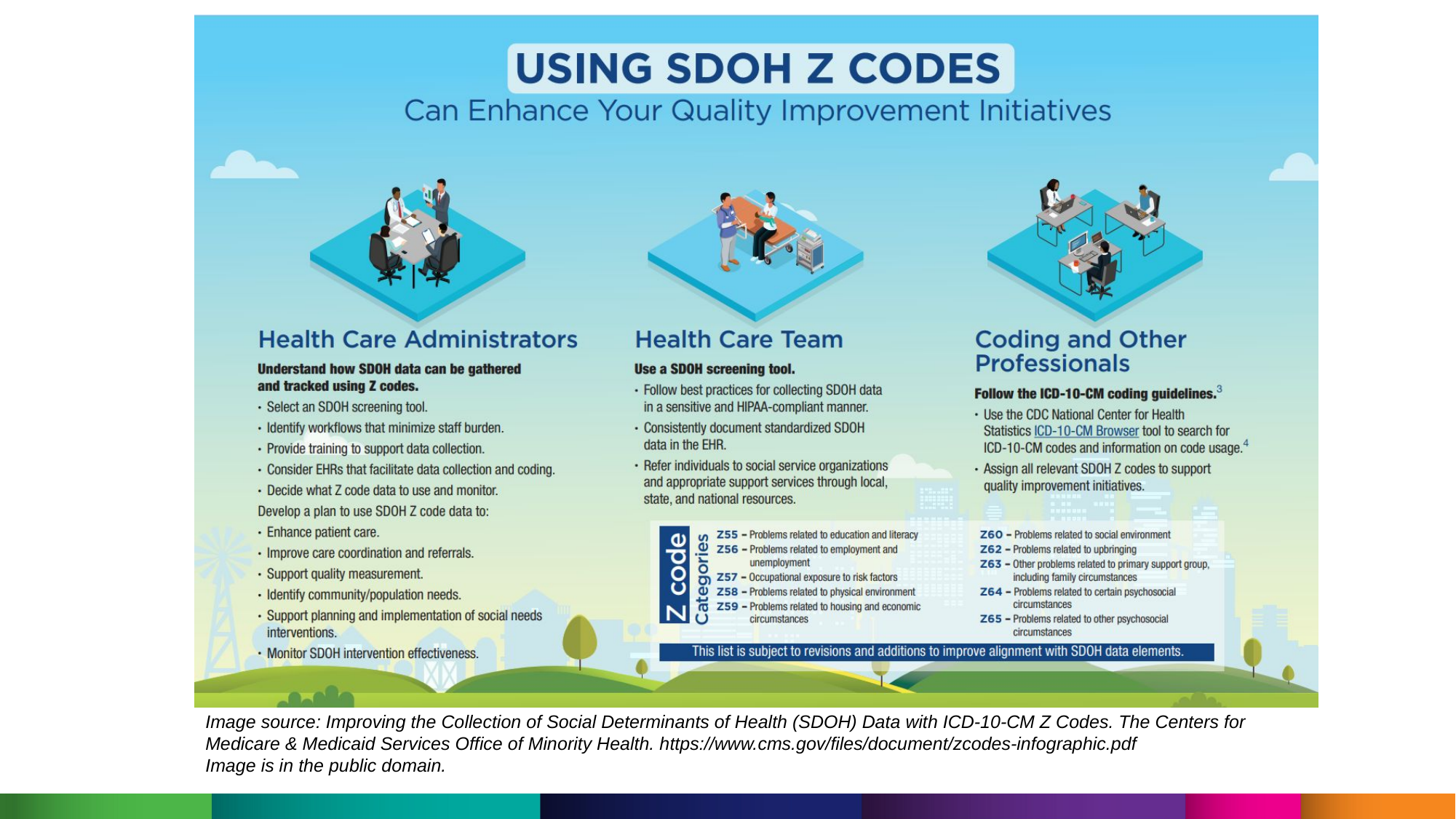

Image source: Improving the Collection of Social Determinants of Health (SDOH) Data with ICD-10-CM Z Codes. The Centers for Medicare & Medicaid Services Office of Minority Health. https://www.cms.gov/files/document/zcodes-infographic.pdf
Image is in the public domain.

## Slide 25
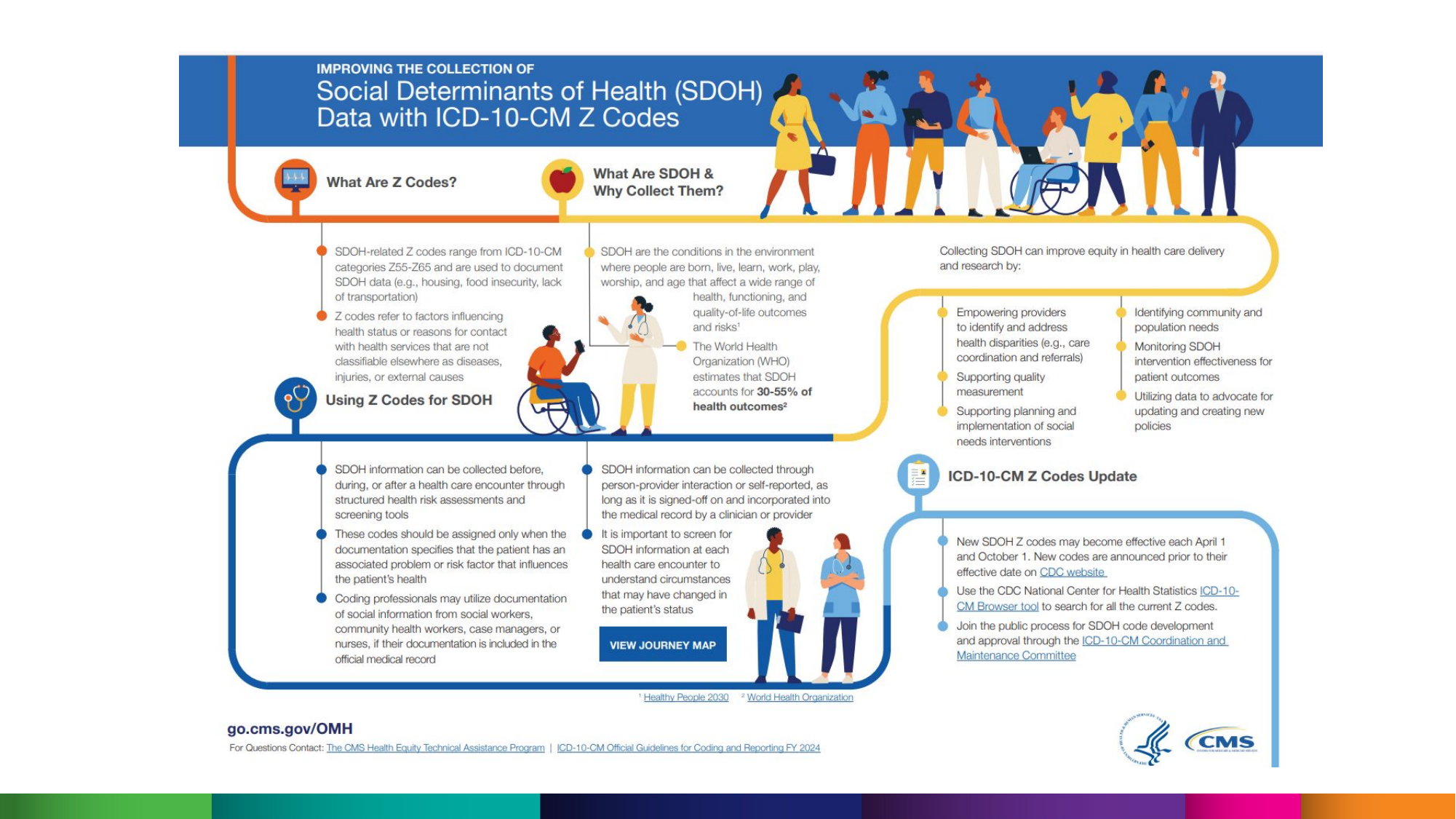

## Slide 26
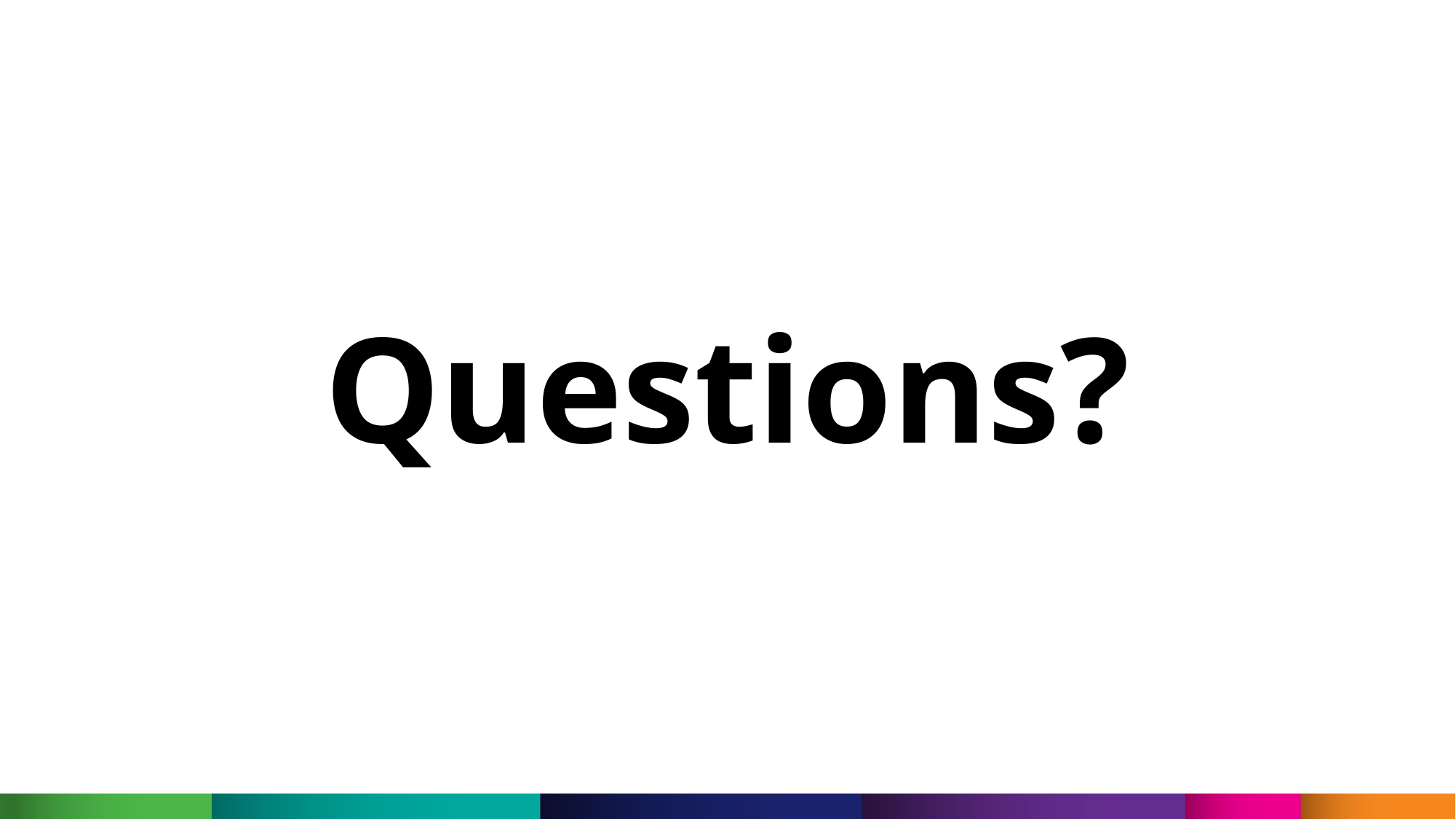

# Questions?

## Slide 27
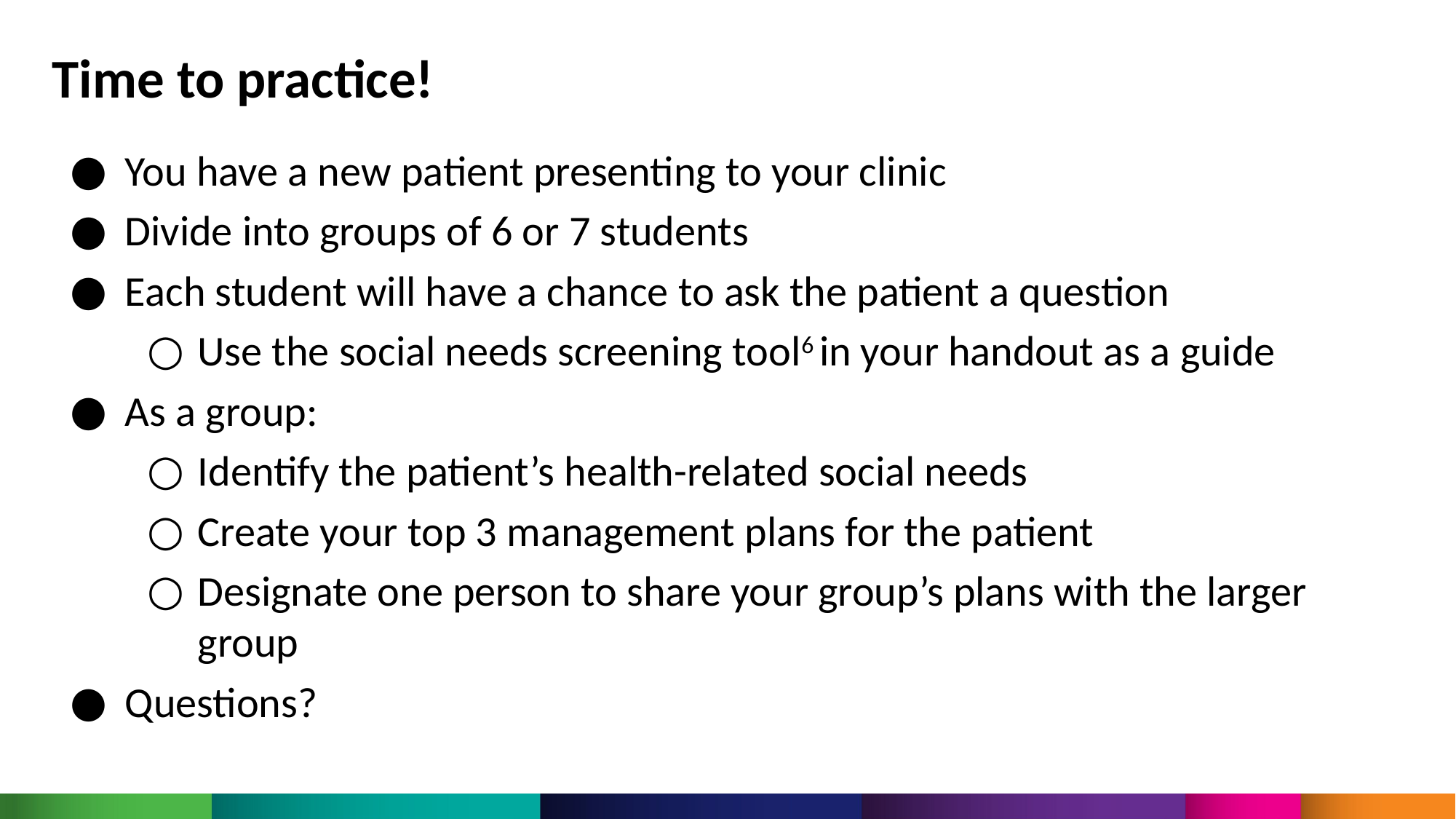

# Time to practice!
You have a new patient presenting to your clinic
Divide into groups of 6 or 7 students
Each student will have a chance to ask the patient a question
Use the social needs screening tool6 in your handout as a guide
As a group:
Identify the patient’s health-related social needs
Create your top 3 management plans for the patient
Designate one person to share your group’s plans with the larger group
Questions?

## Slide 28
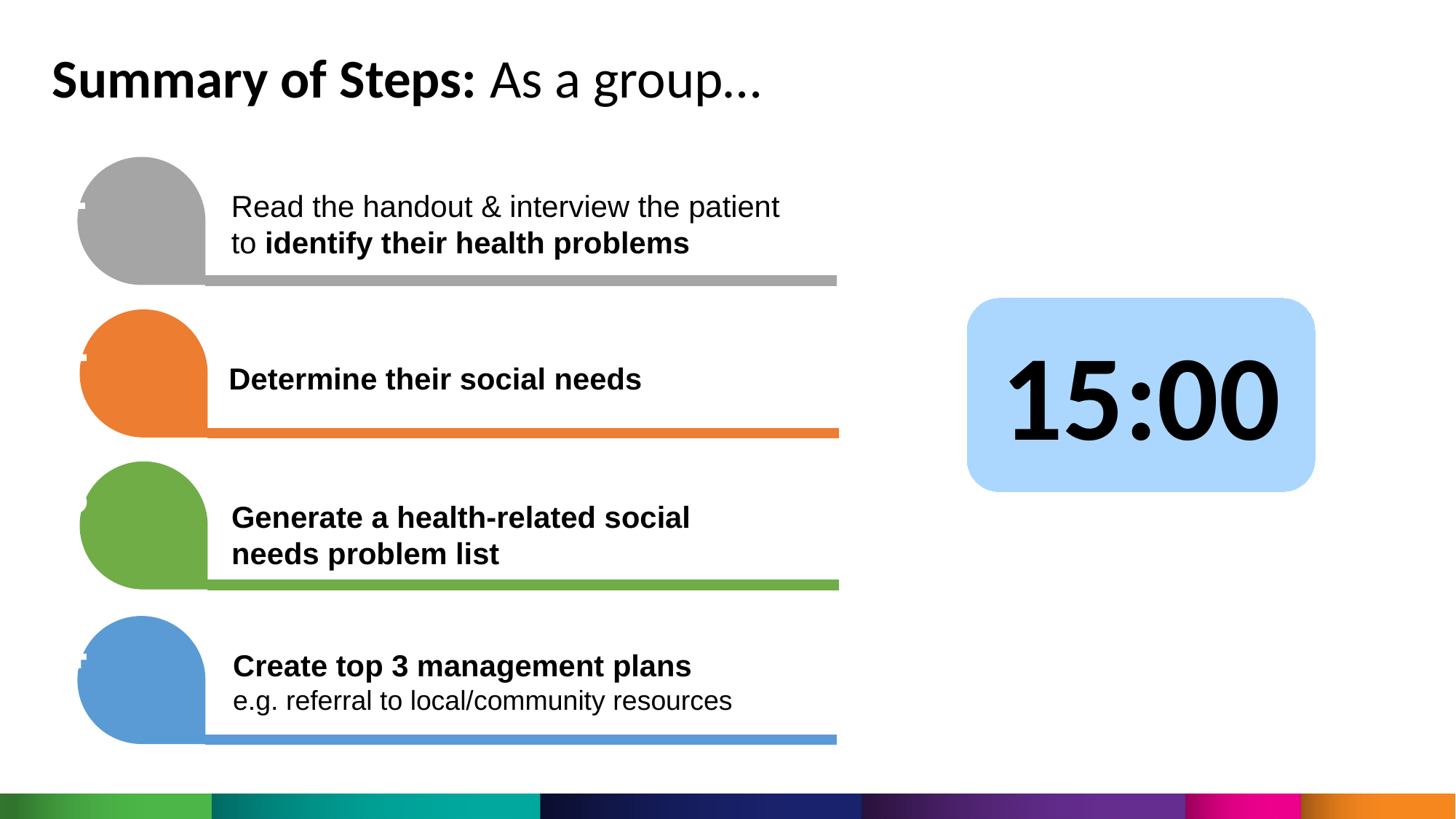

# Summary of Steps: As a group…
1
Read the handout & interview the patient to identify their health problems
2
Determine their social needs
3
Generate a health-related social needs problem list
4
Create top 3 management plans
e.g. referral to local/community resources
15:00

## Slide 29
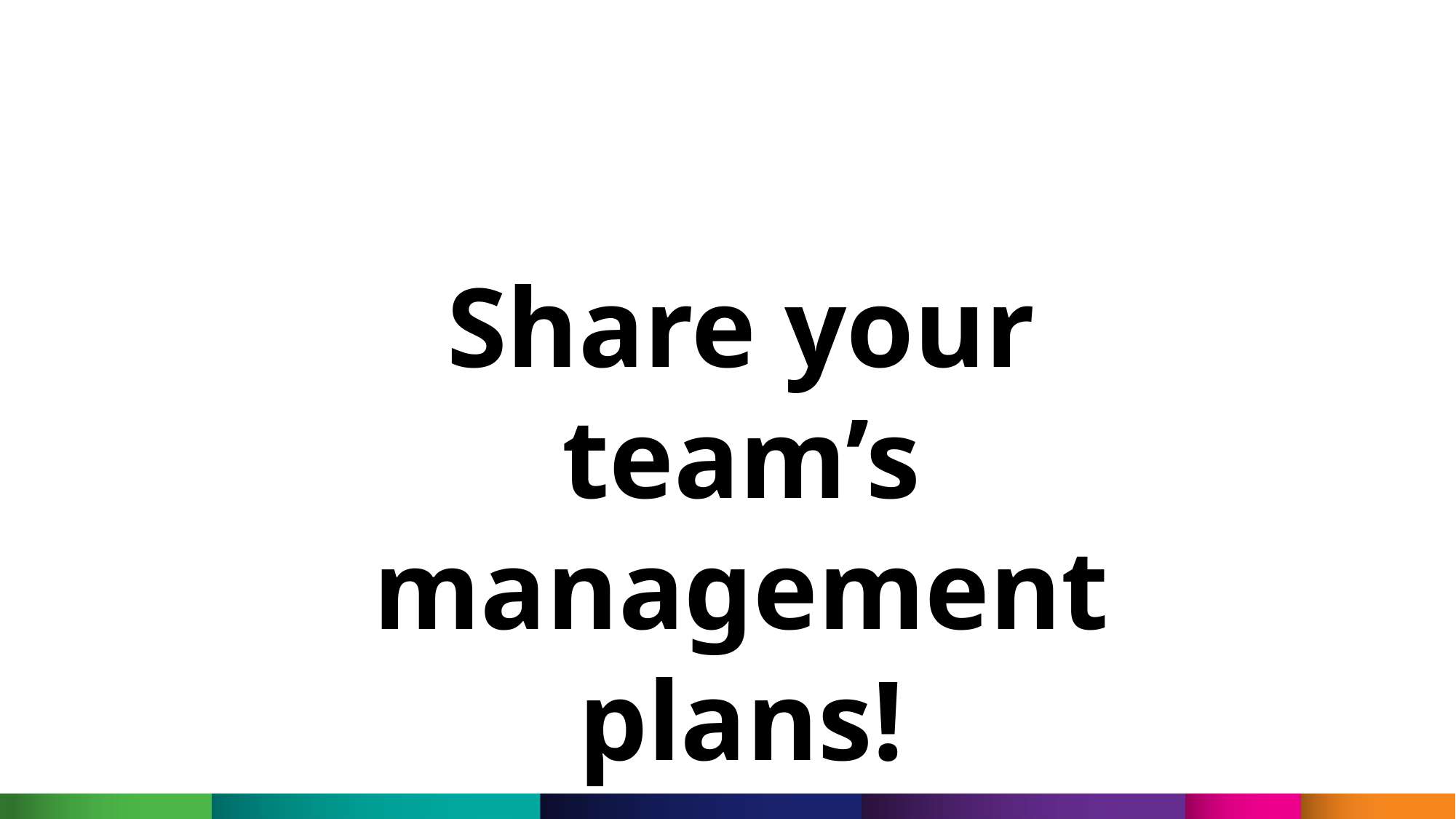

# Share your team’s management plans!

## Slide 30
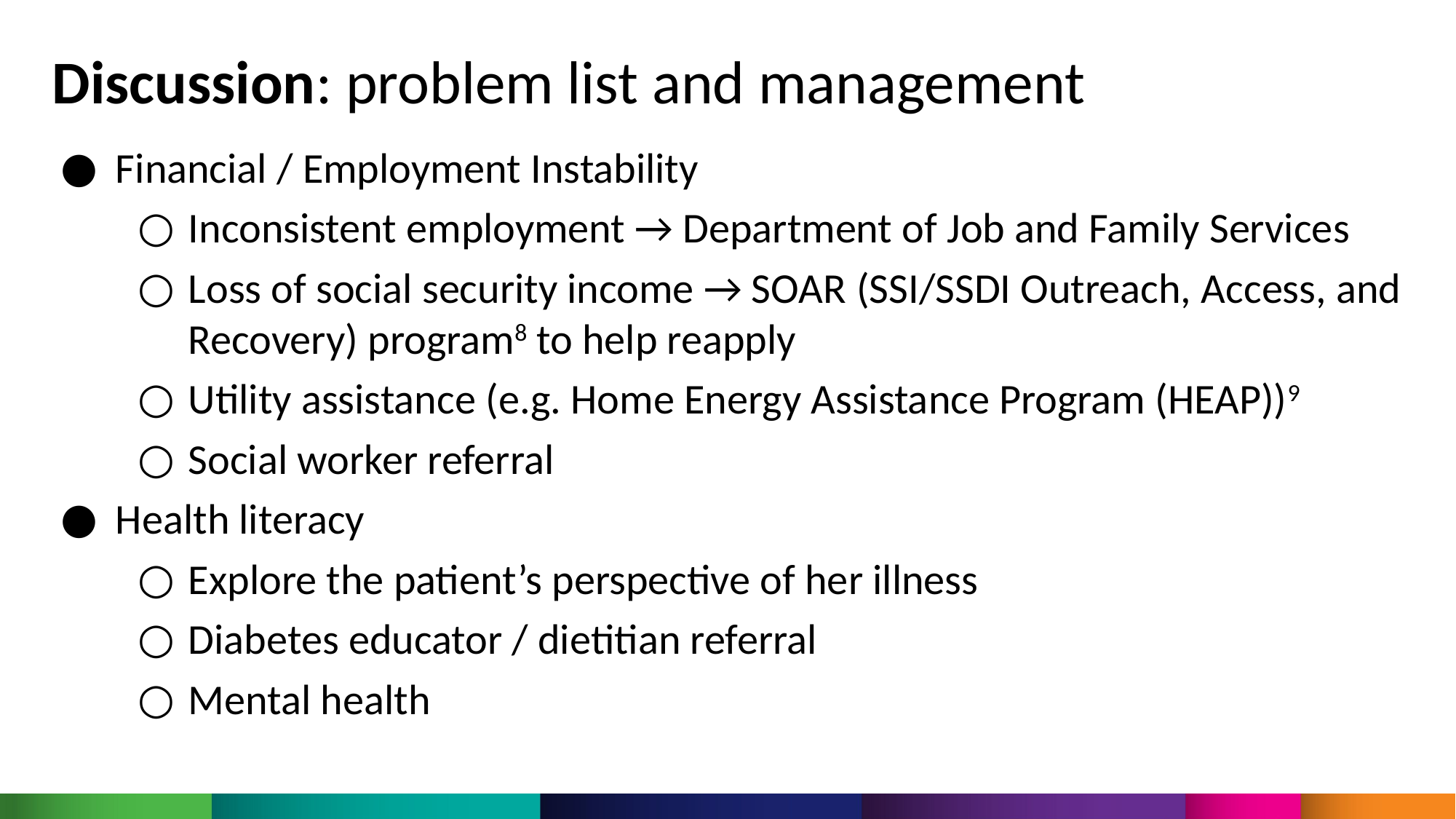

# Discussion: problem list and management
Financial / Employment Instability
Inconsistent employment → Department of Job and Family Services
Loss of social security income → SOAR (SSI/SSDI Outreach, Access, and Recovery) program8 to help reapply
Utility assistance (e.g. Home Energy Assistance Program (HEAP))9
Social worker referral
Health literacy
Explore the patient’s perspective of her illness
Diabetes educator / dietitian referral
Mental health

## Slide 31
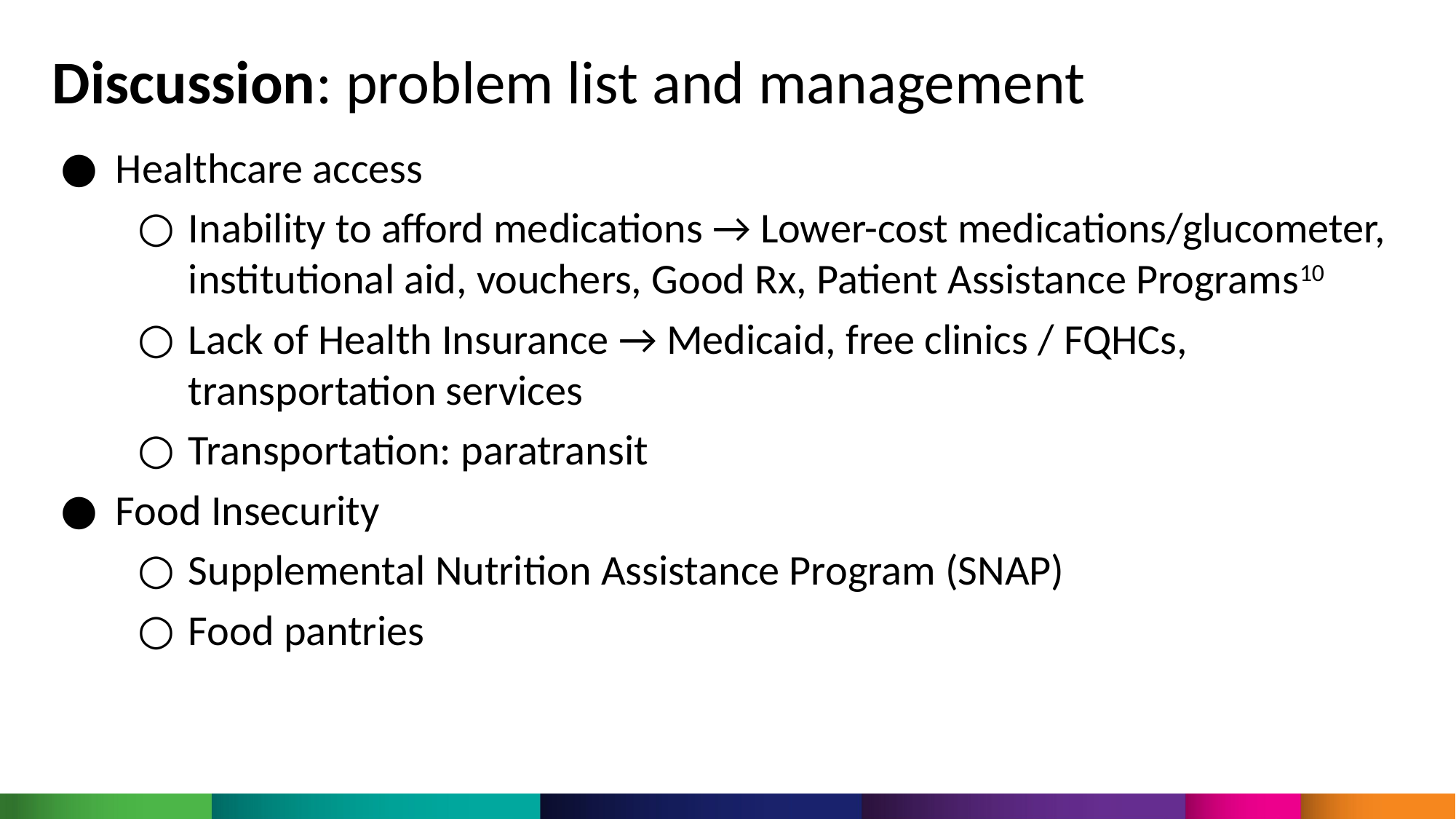

# Discussion: problem list and management
Healthcare access
Inability to afford medications → Lower-cost medications/glucometer, institutional aid, vouchers, Good Rx, Patient Assistance Programs10
Lack of Health Insurance → Medicaid, free clinics / FQHCs, transportation services
Transportation: paratransit
Food Insecurity
Supplemental Nutrition Assistance Program (SNAP)
Food pantries

## Slide 32
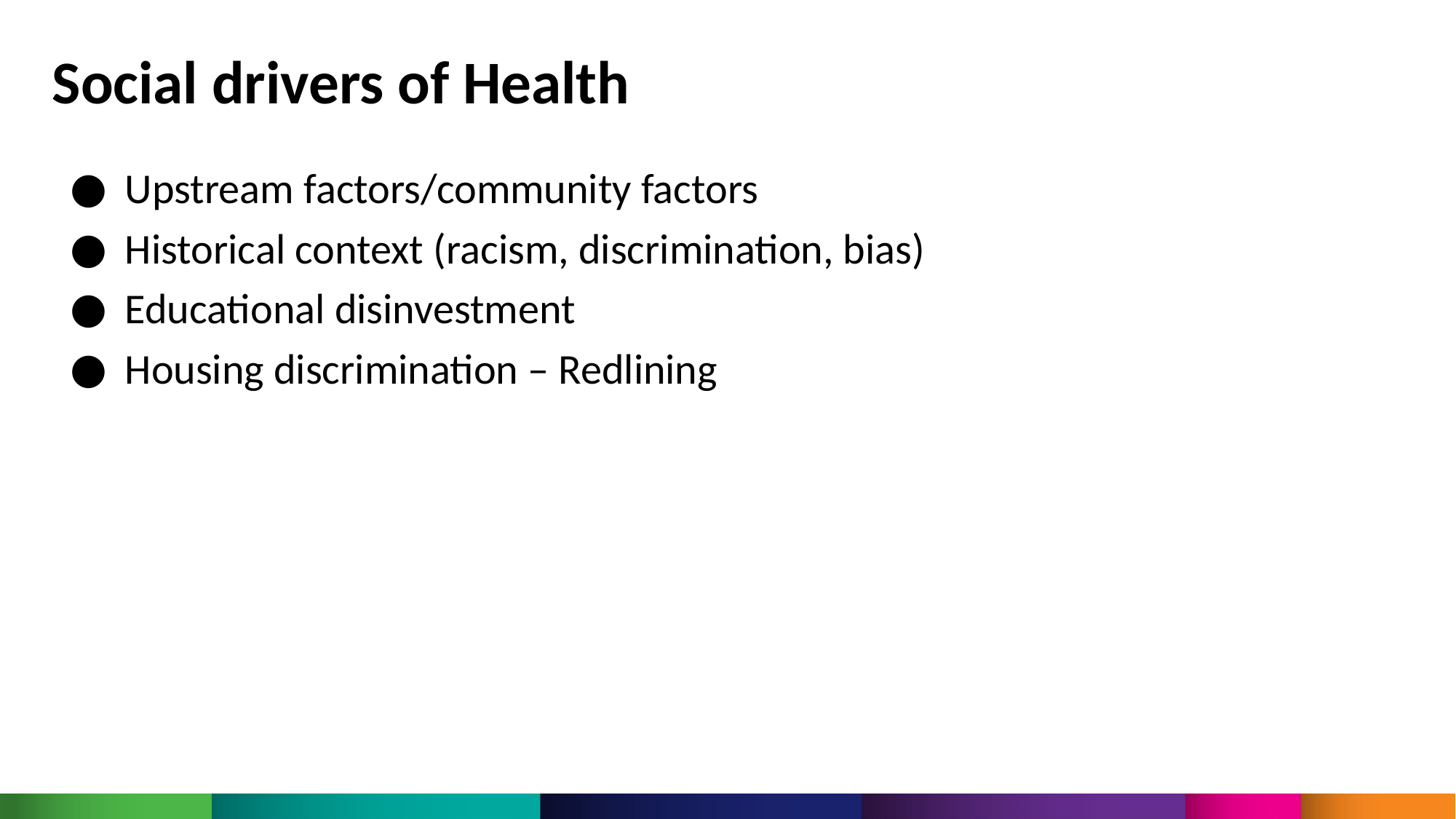

# Social drivers of Health
Upstream factors/community factors
Historical context (racism, discrimination, bias)
Educational disinvestment
Housing discrimination – Redlining

## Slide 33
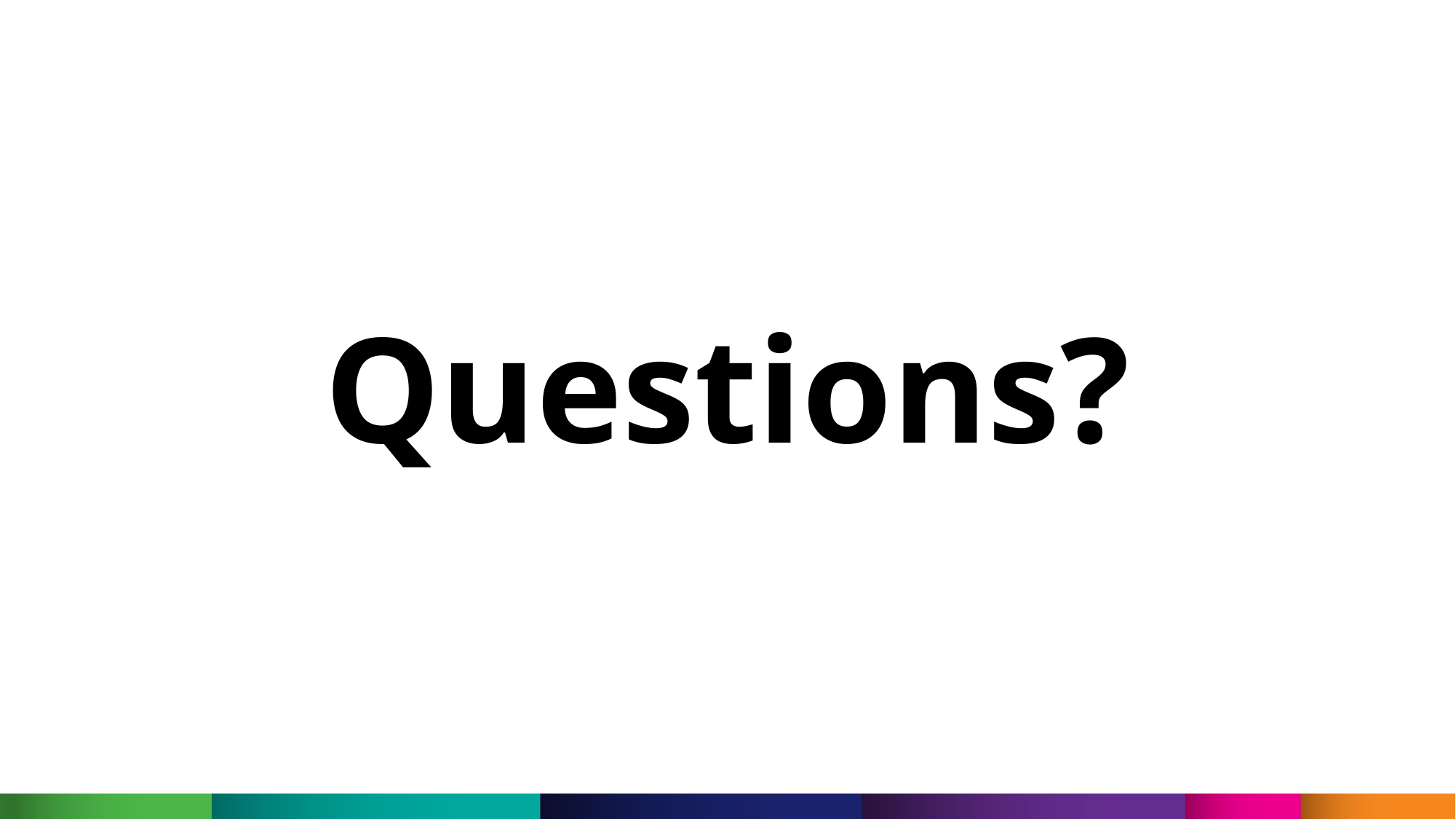

# Questions?

## Slide 34
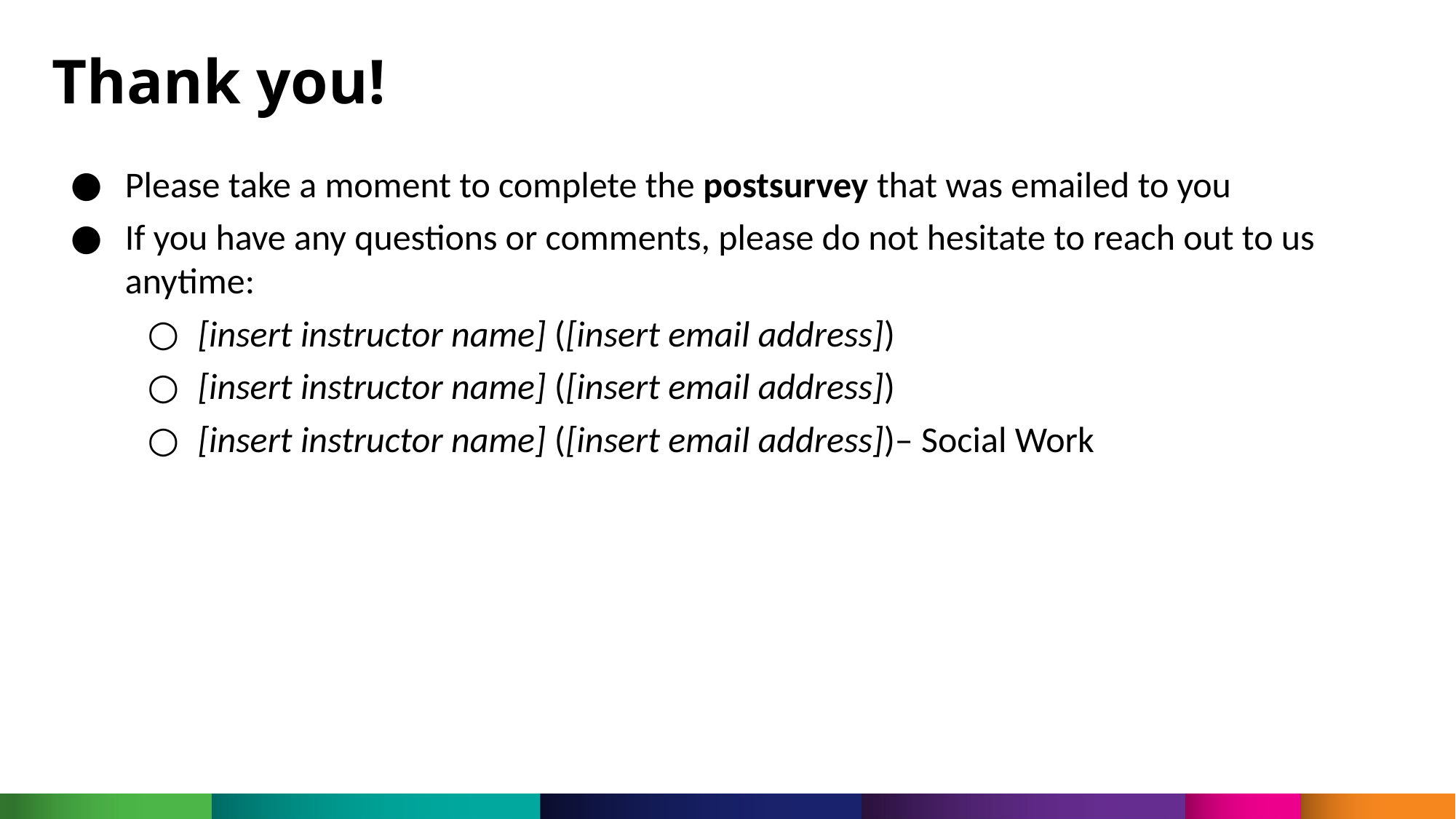

# Thank you!
Please take a moment to complete the postsurvey that was emailed to you
If you have any questions or comments, please do not hesitate to reach out to us anytime:
[insert instructor name] ([insert email address])
[insert instructor name] ([insert email address])
[insert instructor name] ([insert email address])– Social Work

## Slide 35
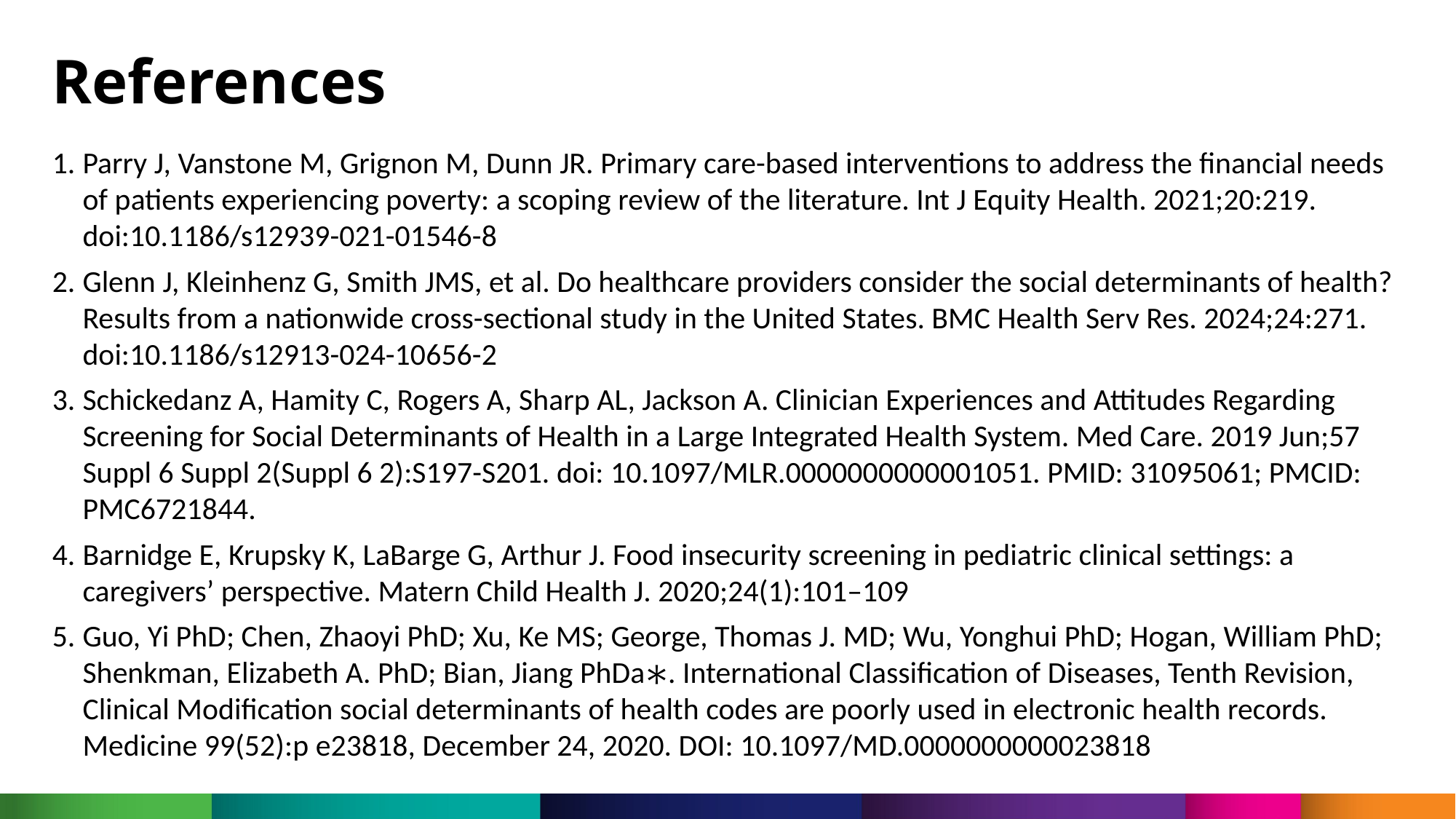

# References
Parry J, Vanstone M, Grignon M, Dunn JR. Primary care-based interventions to address the financial needs of patients experiencing poverty: a scoping review of the literature. Int J Equity Health. 2021;20:219. doi:10.1186/s12939-021-01546-8
Glenn J, Kleinhenz G, Smith JMS, et al. Do healthcare providers consider the social determinants of health? Results from a nationwide cross-sectional study in the United States. BMC Health Serv Res. 2024;24:271. doi:10.1186/s12913-024-10656-2
Schickedanz A, Hamity C, Rogers A, Sharp AL, Jackson A. Clinician Experiences and Attitudes Regarding Screening for Social Determinants of Health in a Large Integrated Health System. Med Care. 2019 Jun;57 Suppl 6 Suppl 2(Suppl 6 2):S197-S201. doi: 10.1097/MLR.0000000000001051. PMID: 31095061; PMCID: PMC6721844.
Barnidge E, Krupsky K, LaBarge G, Arthur J. Food insecurity screening in pediatric clinical settings: a caregivers’ perspective. Matern Child Health J. 2020;24(1):101–109
Guo, Yi PhD; Chen, Zhaoyi PhD; Xu, Ke MS; George, Thomas J. MD; Wu, Yonghui PhD; Hogan, William PhD; Shenkman, Elizabeth A. PhD; Bian, Jiang PhDa∗. International Classification of Diseases, Tenth Revision, Clinical Modification social determinants of health codes are poorly used in electronic health records. Medicine 99(52):p e23818, December 24, 2020. DOI: 10.1097/MD.0000000000023818

## Slide 36
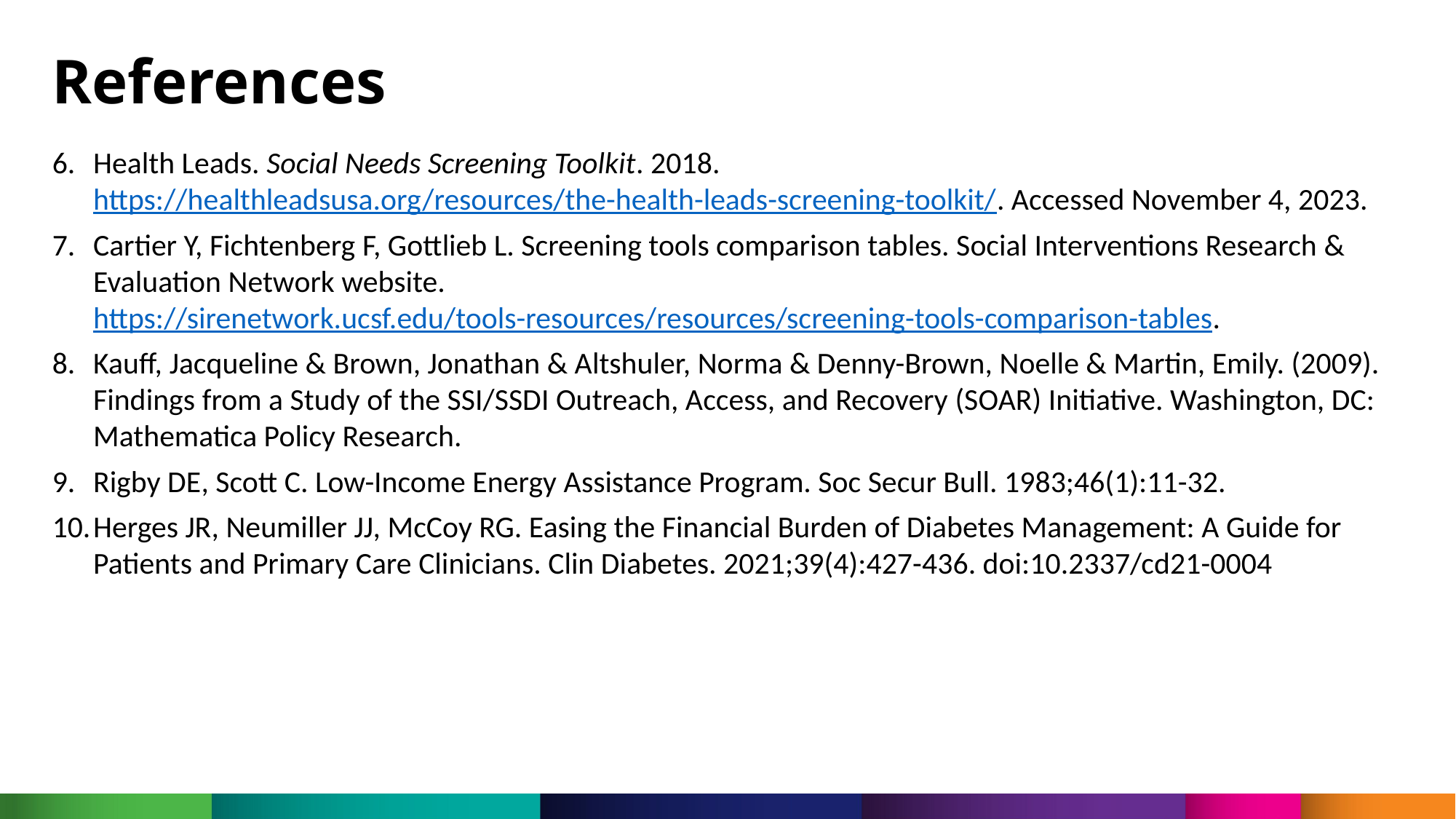

# References
Health Leads. Social Needs Screening Toolkit. 2018. https://healthleadsusa.org/resources/the-health-leads-screening-toolkit/. Accessed November 4, 2023.
Cartier Y, Fichtenberg F, Gottlieb L. Screening tools comparison tables. Social Interventions Research & Evaluation Network website. https://sirenetwork.ucsf.edu/tools-resources/resources/screening-tools-comparison-tables.
Kauff, Jacqueline & Brown, Jonathan & Altshuler, Norma & Denny-Brown, Noelle & Martin, Emily. (2009). Findings from a Study of the SSI/SSDI Outreach, Access, and Recovery (SOAR) Initiative. Washington, DC: Mathematica Policy Research.
Rigby DE, Scott C. Low-Income Energy Assistance Program. Soc Secur Bull. 1983;46(1):11-32.
Herges JR, Neumiller JJ, McCoy RG. Easing the Financial Burden of Diabetes Management: A Guide for Patients and Primary Care Clinicians. Clin Diabetes. 2021;39(4):427-436. doi:10.2337/cd21-0004
