# Supplementary material for: Preparing Future Physicians to Address the Social Needs of Patients in Their Daily Clinical Practice: An Interactive Workshop
Source: MedEdPORTAL. 2026 Apr 21;22:11595. doi: 10.15766/mep_2374-8265.11595 (PMC13098288; doi:10.15766/mep_2374-8265.11595)
Supplement: Supplementary file 1 — Student Handouts.pdfIncorporating SDH Into Patient Care.pptxSmall-Group Case (Student Version).docxSmall-Group Facilitator Training and Full Vignette.docxPresurvey.docxPostsurvey.docx1-Year Follow-Up Survey.docxKnowledge Questions - Answer Key.docx [file mep_2374-8265.11595-s001.zip › C. Small-Group Case (Student Version).docx]

**Appendix C. Small-group Case (Student Version)**

Printed copies of Appendix C were distributed to the students at the beginning of the small-group patient stimulation activity. This appendix contains partial details of the patient’s history (the full history is detailed in the Facilitator Version in Appendix D) such as reasons for the visit and basic demographic information. One of the students’ tasks in this simulation is to identify the remaining biopsychosocial needs of the patient that is contributing to their medical condition by applying the strategies taught in the didactic session and the social drivers of health screening tool (Appendix B) as they interview the patient.

Please see the speaker notes in slide 28 of the presentation PowerPoint (Appendix A) for the suggested time allotment for the activity.

**Small-group patient simulation activity guide (student version)**

| **Demographic Information and the Reason for the Visit** | |
| --- | --- |
| Name (Preferred Name) | Marilyn Peters (Marilyn) |
| Age | 44 years |
| Sex at Birth | Female |
| Preferred pronouns | She/Her/Hers |
| Reason for the visit | Medication refill |
| **Past Medical History and Medications** | |
| Type 2 Diabetes Mellitus | - Diagnosed 3 years ago - Doesn’t monitor her blood glucose levels - Denies polydipsia, polyuria, or hypoglycemic symptoms - Has been taking insulin intermittently |
| Hypertension | - Diagnosed 3 years ago - Has been on 10 mg Lisinopril (ACE inhibitor) every day until 2 weeks ago |
| Left-sided stroke | - One episode 2 years ago with minimal residual defects on daily functioning - Currently taking 81 mg Aspirin |
| **Social History** | |
| - Unemployed, occasionally works in construction - No Tobacco, alcohol or drugs - Poor diet - Exercise: None but walks everywhere | |
| **Physical Exam** | |
| Vitals | - Blood pressure: 160/100 - Heart rate: 80 beats/min - Respiratory rate: 18 breaths/min - Temperature: 97˚F - SpO_2_: 99% (room air) - Weight: 270 lbs today; 300 lbs 1 year ago |
| Neurological | - Right-handed patient - Mild weakness of the left arm and hand - Sensation of the lower extremities are intact bilaterally |

As a group:

1. Spend about 10 minutes to interview Ms. Peters
2. Use the algorithm to develop a health related social needs problem list
3. Devise your diagnosis codes and management plans to address Ms. Peters’ needs
