# Supplementary material for: Preparing Future Physicians to Address the Social Needs of Patients in Their Daily Clinical Practice: An Interactive Workshop
Source: MedEdPORTAL. 2026 Apr 21;22:11595. doi: 10.15766/mep_2374-8265.11595 (PMC13098288; doi:10.15766/mep_2374-8265.11595)
Supplement: Supplementary file 1 — Student Handouts.pdfIncorporating SDH Into Patient Care.pptxSmall-Group Case (Student Version).docxSmall-Group Facilitator Training and Full Vignette.docxPresurvey.docxPostsurvey.docx1-Year Follow-Up Survey.docxKnowledge Questions - Answer Key.docx [file mep_2374-8265.11595-s001.zip › D. Small-Group Facilitator Training and Full Vignette.docx]

**Appendix D. Small-group Facilitator Training and Full Case Details**

Three family physicians—one of the authors (IAA) and her two colleagues—facilitated the small-group simulation activity of the workshop, serving the dual role of both a patient for the patient encounter simulation and a moderator for the discussion portion, when students collaborated to devise biopsychosocial problem lists and management plans.

Prior to the workshop, the facilitators received 30-minute training in person, where we provided them with a brief overview of the goals, audience, design, and the main contents of the workshop.

We shared the following details of the small-group activity:

- Number of students in each group: 6-7 students
- Three components of the activity: patient chart review, patient interview, and group discussion to develop biopsychosocial problem lists and management plans
- Total duration of the small-group activity: 15 minutes
  - We provided a general recommendation to allocate 2 minutes for students to review the patient’s information, 7 minutes for the patient encounter, and 6 minutes for the discussion.

We then covered the details of the patient encounter:

- Setting: outpatient primary care clinic
- Facilitators’ role: a patient presenting to an outpatient clinic for medication refill
- Students’ role (collectively as a group): a primary care physician
- Relevant patient details outlined on pages 3 and 4 of this document (Appendix D)
- Partial details that would be provided to the students (Appendix C); we informed the facilitators that students were not expected to uncover all details.
- Handouts, including the social needs screening tool,^1^ that would be provided to the students (Appendix B)
  - While students were instructed to use the tools as a guide, we did not provide facilitators with scripted answers to the screening questions because the education objective was for students to practice applying the tools according to the clinical context for comprehensive screening, rather than recite the questions.
- As the patient’s responses to the screening questions themselves were not essential to the educational objective, we encouraged the facilitators to answer students’ questions at their discretion based on the case details we provided.

Finally, we informed the facilitators that, when about 6 minutes are remaining, we will make an announcement to prompt students to transition to the group discussion if they had not already done so. We then explained that facilitators would moderate the discussion, maintaining a minimal role to promote student-driven discussion and practice in devising management plans. We asked them to intervene only when discussions stalled or required redirection, using probing questions such as those shown on page 5.

**Small-group patient simulation activity guide (facilitator version)**

| **Demographic Information and the Reason for the Visit** | | |
| --- | --- | --- |
| Name (Preferred Name) | Marilyn Peters (Marilyn) | |
| Age | 44 years | |
| Sex at Birth | Female | |
| Preferred pronouns | She/Her/Hers | |
| Reason for the visit | Medication refill | |
| **Past Medical History and Medications** | | |
| Type 2 Diabetes Mellitus | - Diagnosed 3 years ago - Has not been monitoring her blood glucose levels because she lost her glucometer when she moved one year ago. - Denies polydipsia, polyuria, or hypoglycemic symptoms. - Has been taking insulin intermittently due to difficulty affording the medication. She has been rationing her insulin since her last prescription refill. - Medications – Glargine 20 units at bedtime, Metformin XR 1500, Empagliflozin 25 mg daily | |
| Hypertension | - Diagnosed 3 years ago - Has been taking 10 mg Lisinopril (ACE inhibitor) every day until 2 weeks ago, when she ran out of her medication. | |
| Left-sided stroke | - One episode 2 years ago with minimal residual defects on daily functioning - Currently taking 81 mg Aspirin | |
| **Physical Exam** | | |
| Vitals | - Blood pressure: 160/100 mmHg - Heart rate: 80 beats/min - Respiratory rate: 18 breaths/min - Temperature: 97˚F - SpO_2_: 99% (room air) - Weight: 270 lbs today; 300 lbs 1 year ago - Unintentional weight loss. Has been eating less and skipping meals to reduce her food expenses. Her diet is poor due to cost and she eats fast food regularly. - Point-of-care hemoglobin A1c is 9.6% (average glucose 225) | |
| Neurological | - Mild weakness of the left arm and hand - doesn’t affect daily function. - Sensation of the lower extremities are intact bilaterally | |
| **Social History** | | |
| Occupation | | Occasionally works odd jobs in construction |
| Social support | | Lives with her spouse, who works but not enough to pay the bills |
| Health insurance | | None |
| Diet | | Mostly fast foods or other inexpensive high caloric foods |
| Exercise | | “I walk everywhere” |
| Alcohol, tobacco, or recreational drug use | | None |
| Sexual history | | - Currently sexually active, monogamous with her spouse - Does not use barrier contraception - History of tubal ligation |
| Highest level of education | | High school |
| **Other relevant information** | | |
| - Has not seen a healthcare provider in 2 years - Unemployed. Had a small income from social security which she lost more than 1 year ago due to her odd jobs, she no longer qualified for social security due to the increase in income it caused. She stopped the odd jobs about 1 year ago and has been trying to reapply, but has not been successful. | | |

**Probing Questions (if needed)**

1. What aspects of her medical problems need further examining?
2. What are the barriers to the patient effectively managing her medical problems?
3. What factors could contribute to this?
4. How could you elicit those factors during an interview?
5. What are some resources that are available for those affected by health-related social needs?

The following two questions may be discussed in the large-group session after the small-group exercise (they are provided here for your context).

1. Discuss poverty and its historical context in Cleveland and the United States.
2. How would the social drivers of health affect Marilyn’s ability and motivation to seek care?

**The student should elicit the following health-related social needs problem list:**

- Financial/economic insecurity
- Food insecurity
- Access/lack of insurance
- Housing insecurity

**Some examples of the management plan are:**

- Food as medicine, food bank, Supplemental Nutrition Assistance Program (SNAP)
- Utility assistance (e.g. Home Energy Assistance Program (HEAP))
- Insurance, financial assistance
- Lower-cost medications/glucometer, institutional aid, vouchers, Good Rx, Medicaid, Patient Assistance Programs^2^
- Legal aid, Department of Job and Family Services
- Social work referral
- Diabetes educator / dietitian referral

References

1. Centers for Medicare & Medicaid Services. The Accountable Health Communities Health-Related Social Needs Screening Tool. Published online 2023. Accessed September 3, 2023. https://www.cms.gov/priorities/innovation/files/worksheets/ahcm-screeningtool.pdf

2. Herges JR, Neumiller JJ, McCoy RG. Easing the Financial Burden of Diabetes Management: A Guide for Patients and Primary Care Clinicians. *Clin Diabetes*. 2021;39(4):427-436. doi:10.2337/cd21-0004
