# Supplementary material for: Preparing Future Physicians to Address the Social Needs of Patients in Their Daily Clinical Practice: An Interactive Workshop
Source: MedEdPORTAL. 2026 Apr 21;22:11595. doi: 10.15766/mep_2374-8265.11595 (PMC13098288; doi:10.15766/mep_2374-8265.11595)

Appendix E. Presurvey Questions

Students were asked to complete this survey at the beginning of the workshop.

The survey may be administered electronically or on paper. It may also be conducted in a de-identified manner, provided that a given student's responses to pre-, post-, and 1‑year surveys can be linked to the same student to allow tracking of changes in their responses across time points.

Please see Appendix H for suggested guideline on how to score students’ responses to the knowledge-related questions.

| **Knowledge-related Questions** |
| --- |

1. Which of the following patients may benefit from social work intervention? Please check all that apply:


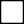
 45-year-old male with osteoarthritis of the knee
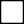
 22-year-old female with depression


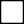
 30-year-old homeless male


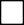
 Pregnant woman at 34 weeks of gestation
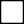
 Child diagnosed with failure to thrive

Please consider the following scenario to answer the next two questions:

A 32-year-old female presents to your clinic for her first prenatal visit for her second child at 32 weeks of gestation. She has a history of substance use disorder, is unemployed, and lives alone. Her first child is in foster care.

1. Which of the following domains of social determinants of health may this patient be affected by?

Please select all that apply:


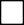
 Economic stability


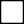
 Education access and quality
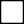
 Healthcare access and quality


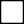
 Neighborhood and built environment
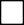
 Social and community context


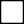
 None of the above

1. Which of the following is/are the most appropriate next step(s) in managing this patient?

Please select all that apply:


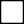
 Active listening


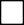
 Referring to a social worker


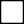
 Recommending a support group
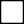
 Calling the police


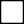
 None of the above

1. After interviewing a patient, you have identified that the patient has an unmet socioeconomic need. Which of the following methods best describes the approach recommended by the Centers for Medicare and Medicaid Services (CMS) to document the identified socioeconomic need, in order to enhance patient care coordination and support future health quality improvement initiatives?
   1. Document the social need in the free-form encounter note field


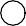

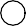

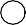

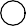

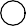


- 1. Enter the social need as a diagnosis code
  2. Send a secure message to the patient's nurse to inform them about the patient's social need
  3. A and C
  4. None of the above

| **Please indicate your level of confidence or comfort in your ability to do each of the following:** |
| --- |

1. Structure your patient interview to effectively elicit sensitive information from the patient regarding their socioeconomic circumstances within the time constraints of a typical encounter
2. Devise a biopsychosocial problem list for a patient
3. Devise a specific management plan that can address a patient's socioeconomic needs
4. Discover what referral resources would be available within a given practice setting (e.g. the healthcare institution that you practice in, the local community) that you would be able to connect your patients to


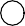

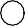

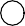

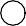

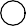


1. Outline the roles of social workers in addressing patients' social needs.


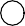

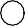

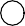

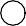

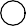


Not at all

confident


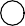


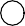


Slightly confident Somewhat


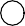

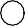

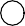

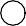

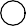


confident


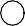

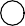


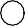

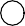


Quite confident Very confident


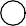

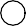


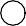

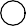


| **Attitude and Behavior Questions**  **For the questions below, please reflect on your interactions with patients in clinical settings or standardized patients and your experiences during clinical cases.** |
| --- |

When interviewing a patient, how often do you ask your patients whether they need any socioeconomic support?

Never

1-25% of the time 26-50% of the time 50-75% of the time

>75% of the time

When constructing a differential diagnosis for a patient's medical problem, how often do you consider socioeconomic causes of their medical condition in your differential diagnosis?

Never

1-25% of the time 26-50% of the time 50-75% of the time

>75% of the time

Please indicate your level of agreement with the following statement:

"I feel hesitant to ask patients about their socioeconomic needs."

Strongly disagree

Disagree

Neither agree nor disagree

Agree

Strongly agree

Please indicate your level of agreement with the following statement: "I feel hesitant to ask patients about their socioeconomic needs."


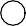

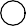

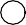

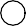

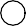

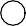

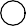

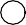

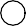

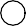

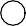

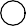

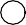

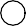

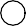

Supplement: Supplementary file 1 — Student Handouts.pdfIncorporating SDH Into Patient Care.pptxSmall-Group Case (Student Version).docxSmall-Group Facilitator Training and Full Vignette.docxPresurvey.docxPostsurvey.docx1-Year Follow-Up Survey.docxKnowledge Questions - Answer Key.docx [file mep_2374-8265.11595-s001.zip › E. Presurvey.docx]
