# Supplementary material for: Preparing Future Physicians to Address the Social Needs of Patients in Their Daily Clinical Practice: An Interactive Workshop
Source: MedEdPORTAL. 2026 Apr 21;22:11595. doi: 10.15766/mep_2374-8265.11595 (PMC13098288; doi:10.15766/mep_2374-8265.11595)
Supplement: Supplementary file 1 — Student Handouts.pdfIncorporating SDH Into Patient Care.pptxSmall-Group Case (Student Version).docxSmall-Group Facilitator Training and Full Vignette.docxPresurvey.docxPostsurvey.docx1-Year Follow-Up Survey.docxKnowledge Questions - Answer Key.docx [file mep_2374-8265.11595-s001.zip › H. Knowledge Questions - Answer Key.docx]

Appendix H. Knowledge Questions - ANSWER KEY

This appendix details the correct answers and suggested method to score students’ responses to the knowledge-related questions in the pre-, post-, and 1-year surveys (Appendices E, F, G, respectively).

Each correctly selected and correctly unselected checkbox was awarded 1 point.

No points were awarded for incorrectly selected or unselected checkboxes.

No points were deducted for incorrect selections.

Each student's total score was divided over the maximum possible score (15 points).

1. Which of the following patients may benefit from social work intervention? Please check all that apply: (5 points)


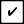
 45-year-old male with osteoarthritis of the knee
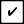
 22-year-old female with depression


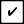
 30-year-old homeless male


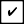
 Pregnant woman at 34 weeks of gestation
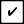
 Child diagnosed with failure to thrive

Please consider the following scenario to answer the next two questions:

A 32-year-old female presents to your clinic for her first prenatal visit for her second child at 32 weeks of gestation. She has a history of substance use disorder, is unemployed, and lives alone. Her first child is in foster care.

1. Which of the following domains of social determinants of health may this patient be affected by? (5 points)

Please select all that apply:


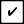
 Economic stability


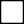
 Education access and quality
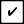
 Healthcare access and quality


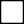
 Neighborhood and built environment
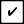
 Social and community context


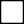
 None of the above (Selecting this box clears any other selection, resulting in a score of 2 out of 5 points)

1. Which of the following is/are the most appropriate next step(s) in managing this patient? (4 points)

Please select all that apply:


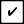
 Active listening


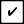
 Referring to a social worker


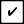
 Recommending a support group
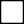
 Calling the police


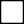
 None of the above (Selecting this box clears any other selection, resulting in a score of 1 out of 4 points)

1. After interviewing a patient, you have identified that the patient has an unmet socioeconomic need. Which of the following methods best describes the approach recommended by the Centers for Medicare and Medicaid Services (CMS) to document the identified socioeconomic need, in order to enhance patient care coordination and support future health quality improvement initiatives? (1 point)
   1. Document the social need in the free-form encounter note field


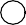

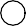

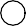

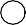


- 1. Enter the social need as a diagnosis code
  2. Send a secure message to the patient's nurse to inform them about the patient's social needs
  3. A and C
  4. None of the above
